# Senescence-like cells recruit γδ T cells to drive prolonged hyposmia after SARS-CoV-2 infection in mice

**DOI:** 10.1038/s44319-026-00769-6

**Published:** 2026-04-10

**Authors:** Shunya Tsuji, Sosuke Nakano, Koyu Ito, Shohei Minami, Ken Uemura, Yusuke Konishi, Masahiro Wakita, Yumiko Okumura, Shimpei Kawamoto, Akari Matsuki, Shinji Nakaoka, Chikako Ono, Hiroo Takahashi, Itsuki Anzai, Tokiko Watanabe, Akiyoshi Uezumi, Yoshiharu Matsuura, Takeshi Kobayashi, Toru Okamoto, Akio Tsuboi, Masataka Asagiri, Eiji Hara

**Affiliations:** 1 Department of Molecular Biology, Research Institute for Microbial Diseases, The University of Osaka, Suita, Japan; 2 Department of Pharmacology, Yamaguchi University Graduate School of Medicine, Ube, Japan; 3 Department of Virology, Research Institute for Microbial Diseases, The University of Osaka, Suita, Japan; 4 HealthSpan Research Center, Tohoku University, Sendai, Japan; 5 Institute of Development, Aging and Cancer, Tohoku University, Sendai, Japan; 6 Organization for Advanced Studies, Tohoku University, Sendai, Japan; 7 Graduate School of Life Science, Hokkaido University, Sapporo, Japan; 8 Laboratory of Virus Control, Research Institute for Microbial Diseases, The University of Osaka, Suita, Japan; 9 Center for Infectious Disease Education and Research, The University of Osaka, Suita, Japan; 10 Department of Molecular Neurobiology, Graduate School of Medicine, Kagawa University, Kagawa, Japan; 11 Department of Molecular Virology, Research Institute for Microbial Diseases, The University of Osaka, Suita, Japan; 12 Medical Institute of Bioregulation, Kyushu University, Fukuoka, Japan; 13 Juntendo University School of Medicine, Tokyo, Japan; 14 Graduate School of Pharmaceutical Sciences, The University of Osaka, Suita, Japan; 15 Immunology Frontier Research Center, The University of Osaka, Suita, Japan

**Keywords:** Immunology, Microbiology, Virology & Host Pathogen Interaction, Molecular Biology of Disease

## Abstract

Persistent hyposmia is a hallmark of post COVID-19 conditions, yet the mechanisms sustaining olfactory dysfunction after viral clearance remain poorly understood. Here, using mouse models of SARS-CoV-2 infection, we show that virus-induced senescence-like changes in uninfected olfactory mucosal fibroblasts persist long after viral clearance and drive prolonged olfactory dysfunction. These senescence-like cells secrete SASP factors, including IFNγ, CXCL9, and CXCL11, thereby recruiting γδ T cells to the olfactory mucosa. The accumulated γδ T cells produce excessive IL-17A, which acts on IL-17 receptor A expressed on olfactory sensory neurons, leading to sustained impairment of their function. Genetic ablation of senescence pathways (p16/p21 double knockout), pharmacological elimination of senescent cells with the senolytic drug ABT263, or olfactory neuron-specific deletion of IL-17 receptor A each significantly alleviate prolonged olfactory dysfunction. These findings identify a senescence–γδ T cell–IL-17A axis as a key driver of prolonged hyposmia following SARS-CoV-2 infection in mice.

## Synopsis

SARS-CoV-2 infection induces persistent senescence-like cells in the olfactory mucosa of mice that sustain olfactory dysfunction after viral clearance. These cells recruit γδ T cells via SASP factors, whose IL-17A production impairs olfactory sensory neuron function.

- SARS-CoV-2 induces senescence-like fibroblasts that persist after viral clearance in the olfactory mucosa of mice.
- Senescence-associated secretory factors recruit γδ T cells, promoting their accumulation in olfactory tissue.
- γδ T cell–derived IL-17A impairs olfactory sensory neuron function and sustains hyposmia.
- Genetic or pharmacological targeting of senescence or IL-17 signaling alleviates olfactory dysfunction.

## Abstract (web summary)

SARS-CoV-2 infection induces persistent senescence-like cells in the olfactory mucosa of mice that sustain olfactory dysfunction after viral clearance. These cells recruit γδ T cells via SASP factors, whose IL-17A production impairs olfactory sensory neuron function.

## Graphical abstract

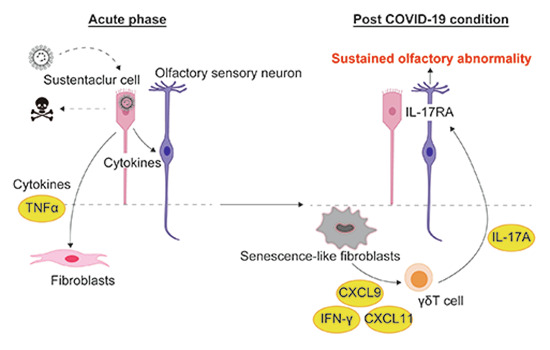

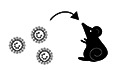

## Introduction

The outbreak of COVID-19, an acute infectious respiratory disease caused by severe acute respiratory syndrome coronavirus 2 (SARS-CoV-2), has recently begun to show signs of abating (Lenharo, 2023). However, approximately 10% of COVID-19 patients exhibit a sequela called post COVID-19 condition (Long COVID) that persists for months to years after the virus is no longer detectable (Peluso and Deeks, 2024; Wulf Hanson et al, 2022). Accordingly, there is an urgent need to elucidate the pathogenetic mechanisms of Long COVID in order to develop effective countermeasures (Akbar and Gilroy, 2020). In the case of hyposmia, a typical manifestation of Long COVID (Zazhytska et al, 2022), studies using human clinical specimens have revealed that IFNγ-secreting γδ T cells infiltrate the olfactory mucosa (OM) even after the virus is no longer detectable in nasal tissue (Finlay et al, 2022), suggesting that persistent inflammatory signalling may contribute to the pathogenesis of this condition. However, it remains unclear why γδ T cells continue to infiltrate the OM in Long COVID and whether their persistence truly contributes to the prolonged olfactory abnormalities (Finlay et al, 2022).

Cellular senescence is a state of irreversible cell-cycle arrest that can be induced by a variety of potentially oncogenic stimuli, and thus is considered to serve as an important tumour suppressor mechanism (Campisi and d’Adda di Fagagna 2007; Gorgoulis et al, 2019). However, since senescent cells do not die immediately and develop an inflammatory phenotype known as the senescence-associated secretory phenotype (SASP) (Acosta et al, 2008; Coppe et al, 2008; Kuilman et al, 2008), the excessive accumulation of senescent cells induces a variety of inflammatory symptoms depending on the biological context (Baz-Martinez et al, 2016; Chan and Narita, 2019, Watanabe et al, 2017). Recently, several groups have reported that SARS-CoV-2 infection induces senescence-like phenomena (Delval et al, 2023; Gioia et al, 2023; Lee et al, 2021; Lipskaia et al, 2022; Schmitt et al, 2023; Tripathi et al, 2021; Tsuji et al, 2022). In particular, we have reported that infection with SARS-CoV-2 also triggers these phenomena in adjacent non-infected cells via paracrine secretion of virus-induced cytokines (Tsuji et al, 2022). Importantly, while most SARS-CoV-2-infected cells die within ~1–2 weeks (Di Domizio et al, 2022; Tsuji et al, 2022), uninfected senescence-like cells are robust and survive for long periods, possibly contributing to the persistence of the inflammatory response via SASP (Tsuji et al, 2022). Furthermore, various senescence markers, including p16^INK4a^ (Hara et al, 1996), have been detected in the nasopharyngeal tissues of COVID-19 patients (Lee et al, 2021; Schmitt et al, 2023). These lines of evidence led us to hypothesize that senescence-like cells induced by SARS-CoV-2 infection may contribute to the persistent olfactory abnormalities observed in Long COVID. Here, using mouse models in which the accumulation of senescent cells can be prevented either genetically or pharmacologically, we show that SARS-CoV-2-induced senescence-like cells persist in the olfactory mucosa after viral clearance and recruit γδ T cells via SASP factors. The γδ T cells in turn secrete IL-17A, which acts on olfactory sensory neurons to sustain prolonged hyposmia.

## Results and discussion

### SARS-CoV-2 infection induces persistent senescence-like cells in the olfactory mucosa

**Figure EV1:**
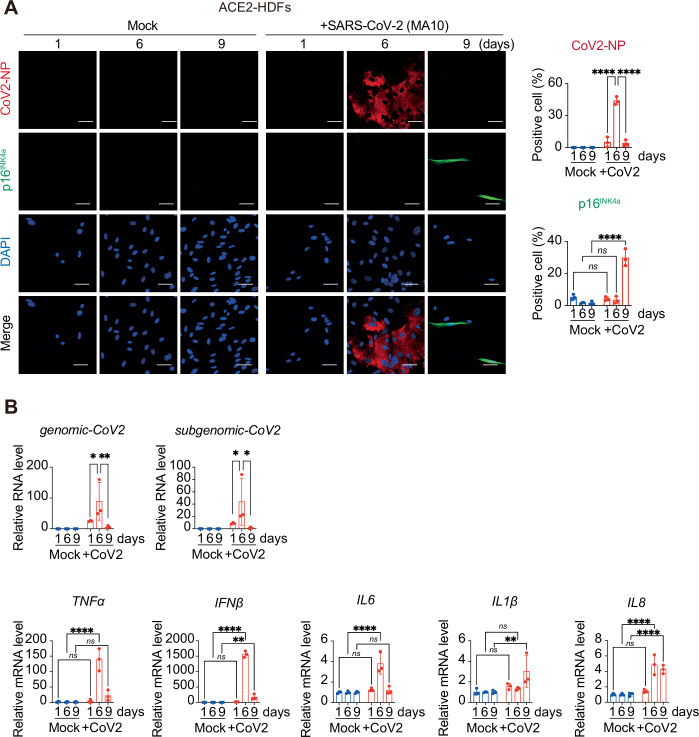
A mouse-adapted SARS-CoV-2 strain induces senescence-like phenotypes in human lung diploid fibroblasts (HDFs). ACE2-HDFs infected with SARS-CoV-2 (MA10 strain; CoV2) or without (Mock) at m.o.i. (multiplicity of infection) of 0.1 were subjected to immunofluorescence analysis (**A**) or to RT–qPCR analysis (**B**) at days 1, 6, and 9. (**A**) Immunofluorescence images of CoV2-NP (red), p16^INK4a^ (green), and DAPI (blue) are shown. The bar graph indicates the percentages of cells expressing CoV2-NP (top) or p16^INK4a^ (bottom). Scale bar, 50 μm. CoV2_NP (****P < 0.0001), p16^INK4a^ (Day 1; *ns*; *P* = 0.949, Day 6; *ns*; *P* = 0.804, Day 9; ****P < 0.0001). (**B**) RT–qPCR analysis of CoV2 RNA, virus- induced cytokines, and SASP factors. Relative mRNA expression levels were determined using the ΔΔCt method after normalization to *GAPDH*. The y-axis shows the relative amount of RNA when the amount of RNA in Mock is set to 1. *Genomic CoV2* (Day 1 vs. Day 6; *P = 0.0262, Day 6 vs. Day 9; **P = 0.0045), *subgenomic CoV2* (Day 1 vs. Day 6; *P = 0.0431, Day 6 va. Day 9; *P = 0.0153), *TNFα* (Day 1; *ns*; *P* = 0.997, Day 6; ****P < 0.0001, Day 9; *ns*; *P* = 0.3915), *IFNβ* (Day 1; *ns*; *P* = 0.988, Day 6; ****P < 0.0001, Day 9; **P = 0.009), *IL6* (Day 1; *ns*; *P* = 0.8785, Day 6; ****P < 0.0001, Day 9; *ns*; *P* = 0.9449), *IL1β* (Day 1; *ns*; *P* = 0.7162, Day 6; *ns*; *P* = 0.9043, Day 9; ***P = 0.0086), and *IL8* (Day 1; *ns*; *P* = 0.7802, Day 6; ****P < 0.0001, Day 9; ****P < 0.0001). For all graphs, data are presented as mean ± standard deviation (s.d.) of biological triplicate measurements. Statistical significance was determined by two-way ANOVA followed by Sidak’s multiple comparison test. *P* values < 0.05 were considered significant. *P < 0.05, **P < 0.01, ***P < 0.001, ****P < 0.0001, ns: not significant. N.D. not detected.

**Figure 1:**
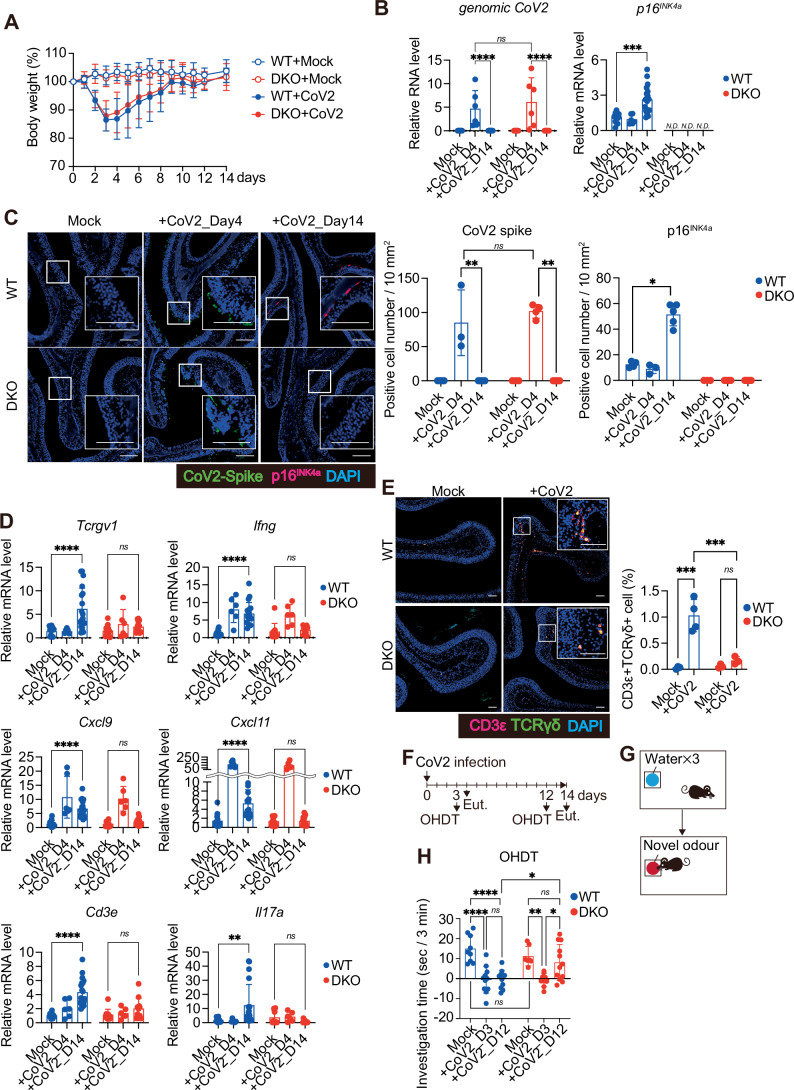
Senescence-like cells induced by SARS-CoV-2 infection prolong inflammatory responses and hyposmia in mice. (**A**–**H**) Ten- to fourteen-week-old female wild-type (WT) mice and p16/p21 double knockout (DKO) mice on a C57BL/6 background were intranasally infected with a mouse-adapted strain of SARS-CoV-2 (CoV2). (**A**) Body weight of inoculated mice was monitored until day 14 post-infection, and is shown as percentage of initial body weight (day 0). Mock (WT, *n* = 9; DKO, *n* = 9), +CoV2 (WT, *n* = 12; DKO, *n* = 10). *n* indicates mice (biological replicates). (**B**) Total RNA was isolated from the olfactory mucosa and subjected to RT–qPCR analysis for CoV2 *genomic RNA* (*genomic CoV2*) and *p16*^*INK4a*^ at the indicating time point. Mock (WT, *n* = 14; DKO, *n* = 11), day 4 (WT, *n* = 6; DKO, *n* = 6) day 14 (WT, *n* = 19; DKO, *n* = 12). Relative mRNA expression levels of *p16*^*INK4a*^ were calculated using the ΔΔCt method, normalised to *β-actin*, and expressed relative to WT Mock (set to 1). Relative RNA levels of *genomic CoV2*, normalised to *β-actin*, are shown. ****P < 0.0001 (*genomic CoV2*), *ns*; *P* = 0.9575 (*genomic CoV2*), and ***P = 0.0001 (*p16*^*INK4a*^). (**C**) Bouin’s-fixed nasal tissues were processed for immunohistochemistry. Representative images showing CoV2 spike protein (green), p16^INK4a^ (red), and DAPI (blue) staining in the olfactory mucosa. Scale bar, 100 μm. Quantification of CoV2 spike-positive and p16^INK4a^-positive cells per 10 mm² of the olfactory mucosa is also shown. Mock (WT, *n* = 4; DKO, *n* = 3), day 4 (WT, *n* = 3; DKO, *n* = 4), day 14 (WT, *n* = 5; DKO, *n* = 5). **P = 0.0083 (CoV2 spike; WT), **P = 0.0095 (CoV2 spike; DKO), *ns*; *P* = 0.6286 (CoV2 spike), and *P = 0.0189 (p16^INK4a^; WT). (**D**) RT–qPCR of the olfactory mucosa was performed for *Tcrgv1*, *Ifng*, *Cxcl9*, *Cxcl11*, *Cd3e*, and *Il17a*, as described in (**B**). Mock (WT, *n* = 14; DKO, *n* = 11), day 4 (WT, *n* = 6; DKO, *n* = 6), day 14 (WT, *n* = 19; DKO, *n* = 12). *Tcrgv1* (****P < 0.0001, *ns*; *P* = 0.8469), *Ifng* (****P < 0.0001, *ns*; *P* = 0.9459), *Cxcl9* (****P < 0.0001, *ns*; *P* = 0.8837), *Cxcl11* (****P < 0.0001, *ns*; *P* = 0.9911), *Cd3e* (****P < 0.0001, *ns*; *P* = 0.2924), and *Il17a* (**P = 0.0013, *ns*; *P* = 0.784). (**E**) Immunofluorescence images of nasal tissue on day 14 post CoV2 inoculation ( + CoV2) or Mock. CD3ε (red), TCRγδ (γδ T cell) (green), and DAPI (blue). Mock (WT, *n* = 3; DKO, *n* = 3), +CoV2 (WT, *n* = 4; DKO, *n* = 3). The proportion of the CD3ε^+^TCRγδ^+^ cells in total DAPI-positive cells in the olfactory mucosa is shown. Scale bar, 50 μm. ***P = 0.0002 (WT), *ns*; *P* = 0.8608 (DKO), and ***P = 0.0005 (WT+CoV2 vs. DKO+CoV2). (**F**–**H**) The olfactory habituation–dishabituation test (OHDT) was performed on day 3 or day 12 after CoV2 inoculation. To avoid confounding due to learning, each mouse was used only once. (**F**) Timeline of the experiments. Euthanasia (Eut.) (**G**) Schematic illustration of the behavioural assay. (**H**) OHDT results using eugenol as a novel odorant. Investigation time is presented as a bar graph. Mock (WT, *n* = 9; DKO, *n* = 7), day 3 (WT, *n* = 11; DKO, *n* = 9), day 12 (WT, *n* = 11; DKO, *n* = 12). ****P < 0.0001 (WT), ns; *P* > 0.9999 (WT), *P = 0.0339 (DKO), **P = 0.0062 (DKO), *ns*; *P* = 0.8981 (DKO), *P = 0.0318 (+CoV2_D12:WT vs. +CoV2_D12:DKO), and *ns*; *P* = 0.8589 (Mock:WT vs. Mock:DKO). All data are presented as mean ± standard deviation (s.d.). Statistical significance was determined by two-way analysis of variance (ANOVA) followed by Sidak’s multiple comparison test (**A**, **B**; *genomic CoV2*, **D**, **E**), One-way ANOVA followed by Tukey’s multiple comparison test (**B**; *p16*^*INK4a*^), nonparametric Kruskal–Wallis test followed by Dunn’s multiple comparisons test (**C**), or two-way ANOVA followed by Tukey’s multiple comparison test (**H**). For the statistical analysis of CoV2-spike in Fig. 1C, WT and p16/p21 DKO mice were compared at day 4 using the Mann–Whitney test. *P* values < 0.05 were considered significant. *P < 0.05, **P < 0.01, ***P < 0.001, ****P < 0.0001, ns: not statistically significant. N.D. not detected. Source data are available online for this figure.

**Figure EV2:**
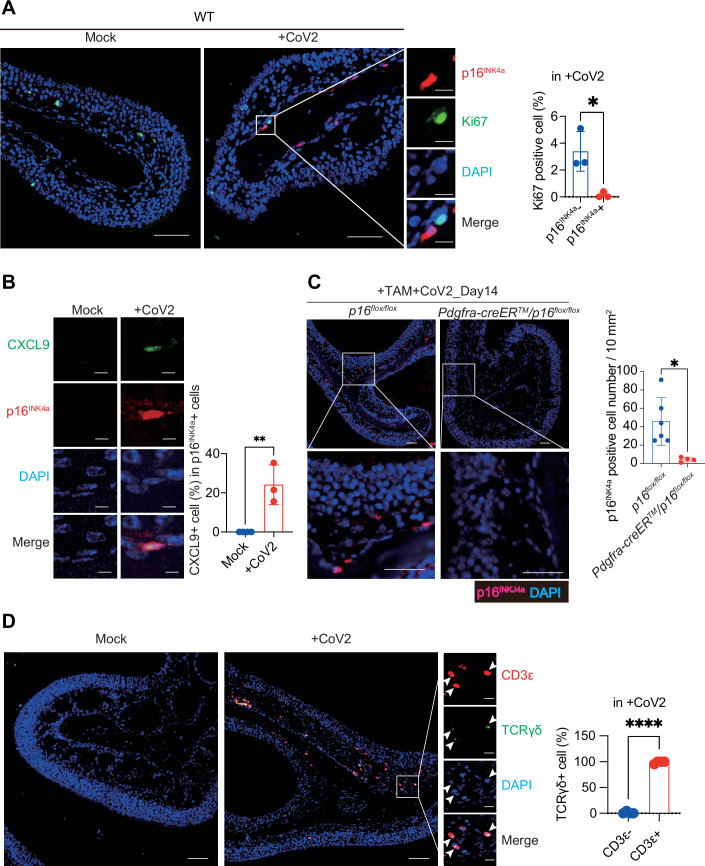
Appearance of senescence-like fibroblasts and infiltration of γδ T cells in the olfactory mucosa after SARS-CoV-2 infection. Ten-week-old female wild-type (WT) C57BL/6 mice were intranasally infected with a mouse-adapted strain of SARS-CoV-2 (CoV2). On day 14 post-infection, nasal tissues were collected, fixed in Bouin’s solution, and decalcified in 10% EDTA buffer (pH 7.0). After paraffin embedding, 5-μm tissue sections were stained (**A**, **B**). (**A**) Representative images showing Ki67 (green; a marker of proliferating cells), p16^INK4a^ (red), and DAPI (blue). The graph shows the frequency of Ki67⁺ cells among p16^INK4a^-negative and -positive cells located beneath the lamina propria, across the entire field of the olfactory mucosa. Scale bars, 50 μm (overview) and 10 μm (higher magnification). *n* = 3. *n* indicates mice (biological replicates). *P = 0.0196. (**B**) Representative images showing CXCL9 (green), p16 (red), and DAPI (blue). The graph shows the percentage of CXCL9-positive cells among total p16-positive cells in the olfactory mucosa. Scale bar: 5 μm. **P = 0.0044. (**C**) Fibroblast-specific *p16* knockout mice (*Pdgfrα-creER*^*TM*^*/p16*^*flox/flox*^, *n* = 4) and *p16*^*flox/flox*^ mice (*n* = 6) were treated with tamoxifen ( + TAM) and inoculated intranasally with mouse-adapted strain of SARS-CoV-2 (CoV2), when mice were 23 weeks old. On day 14 post-infection, nasal tissues were collected, fixed in Bouin’s solution, and decalcified in 10% EDTA buffer (pH 7.0). After paraffin embedding, 5-μm tissue sections were stained for p16^INK4a^ (red) and DAPI (blue). Scale bar, 50 μm. Quantification of p16^INK4a^-positive cells per 10 mm^2^ of the olfactory mucosa is shown. *P = 0.0139. (**D**) Ten-week-old female wild-type (WT) C57BL/6 mice were intranasally infected with a mouse-adapted strain of SARS-CoV-2 (CoV2). On day 14 post-infection, nasal tissues were collected and stained with antibodies against CD3ε (red) and TCRγδ (green), and counterstained with DAPI (blue) to visualise nuclei. Representative immunofluorescence images demonstrate the co-localization of TCRγδ and CD3ε signals in the olfactory mucosa. Arrowheads indicate TCRγδ⁺ cells. The accompanying graph shows the frequency of TCRγδ-positive cells among CD3ε-negative and CD3ε-positive cells in the olfactory mucosa. Scale bars, 50 μm (overview) and 10 μm (higher magnification). *n* = 4 mice. ****P < 0.0001. In all graphs, data represent mean ± standard deviation (s.d.). Statistical significance was determined using an unpaired *t* test. *P* values < 0.05 were considered significant. *P < 0.05, **P < 0.01, ****P < 0.0001.

To examine the roles of senescence-like cells in SARS-CoV-2-induced persistent olfactory abnormalities, we employed murine models in which the accumulation of senescent cells could be prevented either genetically or pharmacologically. We first confirmed that MA10, a mouse-adapted strain of SARS-CoV-2 (Leist et al, 2020; Torii et al, 2021), induces senescence-like phenotypes in adjacent uninfected cells through cytokine secretion from infected cells (Fig. EV1), similar to the SARS-CoV-2 strains causing community infections (Tsuji et al, 2022). Wild-type (WT) mice and p16/p21-double knockout (DKO) mice (Kawamoto et al, 2023), which are less prone to cellular senescence due to the deletion of the important senescence-inducing genes *p16*^*INK4a*^ and *p21*^*Waf1/Cip1/Sdi1*^ (Hara et al, 1996; Noda et al, 1994), were infected with SARS-CoV-2. Notably, both mouse strains displayed similar weight loss (Fig. 1A) and SARS-CoV-2 was detected in the OM (Fig. 1B,C), reaching a peak on the fourth day post-infection. In contrast, although SARS-CoV-2 was no longer detected in the OM at 14 days post-infection, cells expressing high levels of p16^INK4a^ appeared in the OM of WT but not DKO mice (Fig. 1B,C), and these cells were negative for the proliferation marker Ki67 and positive for SASP (Fig. EV2A,B). It is noteworthy that in fibroblast-specific *p16*^*INK4a*^ knockout mice (*Pdgfra-creER*^*TM*^*/p16*^*flox/flox*^ mice) (Kang et al, 2010; Liu et al, 2011), p16^INK4a^ expression was only marginally detected following SARS-CoV-2 infection (Fig. EV2C), suggesting that fibroblasts are the primary cell type responsible for the senescence-like phenotypes observed in the OM under these conditions. These results, together with the observation that sustentacular cells but not fibroblasts are infected by SARS-CoV-2 in the OM (Chen and Wang, 2023), suggest that the senescence-like phenotypes in OM fibroblasts are likely induced indirectly by SARS-CoV-2-infected cells.

### Senescence-like cells promote γδ T cell accumulation in the olfactory mucosa via SASP

Long COVID patients with hyposmia reportedly showed the persistent infiltration of immune cells, especially IFNγ-expressing γδ T cells, into the OM (Finlay et al, 2022). We therefore asked whether the senescence-like cells identified above might be responsible for sustaining this immune cell infiltration. Consistent with this idea, the expression levels of genes encoding the γδ T cell marker, TCR Vγ1 (Pellicci et al, 2020), and IFNγ were increased in the OM of WT, but not DKO, mice at 14 days after SARS-CoV-2 infection (Fig. 1D). Furthermore, this was accompanied by the increased expression of genes encoding CXCL9 and CXCL11, SASP factors involved in γδ T cell recruitment, in the OM of WT mice but not DKO mice (Fig. 1D). It is also important to note that on day 4 post-infection, comparable levels of *Cxcl9, Cxcl11*, and *Ifng* were induced in both WT and DKO mice (Fig. 1D). However, since p16^INK4a^ expression had not yet been upregulated in WT mice at this stage (Fig. 1B,C), these cytokines likely reflect an early antiviral immune response rather than the SASP. Moreover, the expression levels of T cell receptor gamma variable 1 (*Tcrgv1)* also remained low in both groups at this point (Fig. 1D). In contrast, on day 14 post-infection, the expression levels of *Cxcl9*, *Cxcl11*, and *Ifng* remained relatively high in WT mice but were significantly reduced in DKO mice (Fig. 1D), suggesting that the sustained expression of these cytokines is associated with the SASP. At the same time point, the expressions of *Tcrgv1* and the general T cell marker *Cd3e* were elevated in WT mice but not in DKO mice, indicating the accumulation of γδ T cells specifically in WT mice (Fig. 1D). This observation was further supported by an increased number of TCRγδ-positive cells in the OM of WT but not DKO mice (Fig. 1E). These TCRγδ-positive cells also expressed CD3e (Fig. EV2D), confirming the accumulation of γδ T cells at this time point. Collectively, these findings support the hypothesis that SASP-associated CXCL9 and/or CXCL11, secreted by SARS-CoV-2-induced senescence-like cells, sustain local inflammation by promoting the retention of infiltrating γδ T cells in the OM, thereby contributing to prolonged olfactory dysfunction.

### Prolonged olfactory dysfunction correlates with senescence-like cell accumulation

**Figure EV3:**
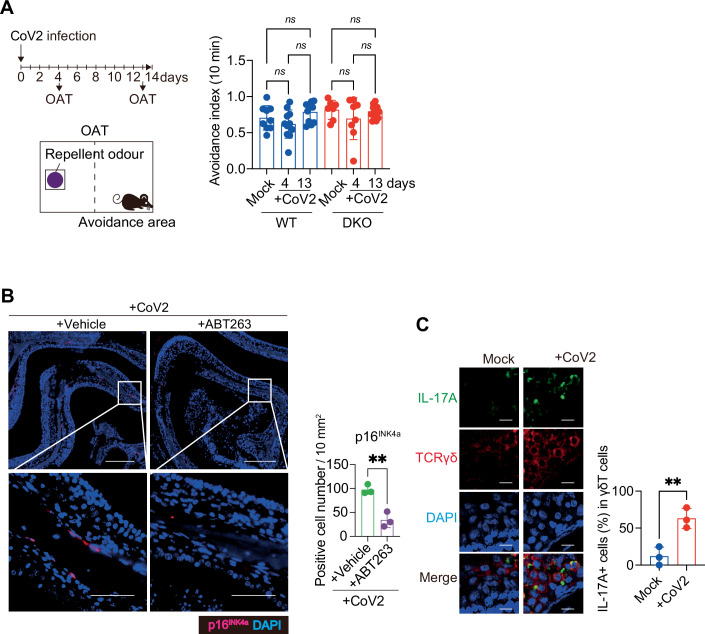
Olfactory avoidance behavior upon p16⁺ cell depletion and validation of IL-17A-secreting γδ T cells in SARS-CoV-2-infected mice. (**A**) Ten-week-old female wild-type (WT) C57BL/6 mice or DKO mice were intranasally infected with a mouse-adapted strain of SARS-CoV-2 (CoV2), and an olfactory avoidance test (OAT) was conducted using 2,4,5-trimethylthiazole (nTMT) as a repellent odour. Avoidance behaviour was quantified as the time spent in the half of the cage opposite the odorant compartment (avoidance area). The avoidance index was calculated as: ([percentage of time in avoidance area during 10 min–50]/50). To avoid learning-related bias, each mouse was used only once in the test. Top left: Timeline of the experimental procedures. Bottom left: Schematic diagram of the OAT setup. Right panel: OAT results. Group sizes: WT Mock (*n* = 9), day 4 (*n *= 11), day 13 (*n* = 11); DKO Mock (*n* = 7), day 4 (*n* = 8), day 13 (*n* = 12). *n* indicates mice (biological replicates). *ns*, WT; *P* = 0.8811 (Mock vs. +CoV2_D4), *P* = 0.9041 (Mock vs. +CoV2_D13), *P* = 0.2355 (+CoV2_D4 vs. +CoV2_D13), DKO; *P* = 0.7453 (Mock vs. +CoV2_D4), *P* = 0.9998 (Mock vs. +CoV2_D13), *P* = 0.7989 (+CoV2_D4 vs. +CoV2_D13). (**B**) Ten-week-old female wild-type (WT) C57BL/6 mice were intranasally infected with a mouse-adapted strain of SARS-CoV-2 (MA10). ABT263 (100 mg/kg) or vehicle was administered by oral gavage at the indicated time points. On day 14 post-infection, nasal tissues were collected, fixed in Bouin’s solution, and decalcified in 10% EDTA buffer (pH 7.0). After paraffin embedding, 5 μm tissue sections were stained for p16^INK4a^ (red) and counterstained with DAPI (blue). The graph shows the number of p16^INK4a^-positive cells per 10 mm² of the olfactory mucosa. Scale bars, 200 μm (overview) and 50 μm (higher magnification). **P = 0.0043. (**C**) Ten-week-old female wild-type (WT) C57BL/6 mice were intranasally infected with a mouse-adapted strain of SARS-CoV-2 (MA10), and nasal tissues were collected at 14 days post-infection. Representative images showing IL-17A (green), TCRγδ (γδ T cells, red), and DAPI (blue). The graph shows the percentage of IL-17A-positive cells among total TCRγδ-positive cells. Scale bar, 10 μm. **P = 0.0086. All data are presented as mean ± standard deviation (s.d.). Statistical significance was determined by two-way ANOVA with Tukey’s multiple comparisons test (**A**) or two-tailed unpaired *t* test (**B**, **C**). *P* values < 0.05 were considered significant. **P < 0.01, ns: not significant.

**Figure 2:**
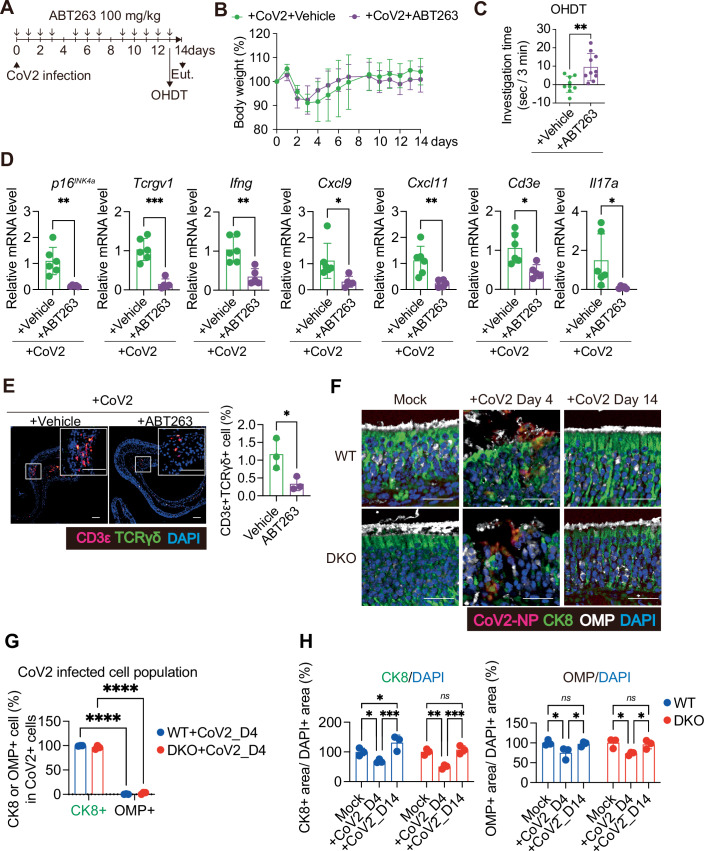
Pharmacological clearance of senescence-like cells attenuates prolonged hyposmia in mice. (**A**–**E**) Ten- to twelve-week-old female WT mice were intranasally inoculated with SARS-CoV-2 (MA10 strain; CoV2), and treated with ABT263 or vehicle at the indicated time points. The olfactory habituation–dishabituation test (OHDT) was performed 8–12 h after the final administration on day 13. (**A**) Timeline of the experiment. ABT263 (100 mg/kg) or vehicle was administered orally on days 0–3, 5–7, and 9–13 post-inoculation. Euthanasia (Eut.) (**B**) Body weight was monitored until day 14 post-CoV2 inoculation, and is shown as percentage of initial body weight (day 0). CoV2+vehicle (*n* = 9), CoV2 + ABT263 (*n* = 10). *n* indicates mice (biological replicates). (**C**) OHDT was performed on day 13 using eugenol as a novel odorant. To avoid confounding due to learning, each mouse was used only once. Investigation time is presented as a bar graph. CoV2+vehicle (*n* = 9), CoV2 + ABT263 (*n *= 10). **P = 0.0043. (**D**), RT–qPCR of the olfactory mucosa was performed for *p16*^*INK4a*^, *Tcrgv1*, *Ifng*, *Cxcl9*, *Cxcl11*, *Cd3e*, and *Il17a*. CoV2+vehicle (*n* = 6), CoV2 + ABT263 (*n* = 5). Relative mRNA expression levels were determined using the ΔΔCt method after normalisation to *β-actin*. Expression levels were normalised to CoV2+vehicle (set to 1). *p16*^*INK4a*^ (**P = 0.003), *Tcrgv1* (***P = 0.0001), *Ifng* (**P = 0.0024), *Cxcl9* (*P = 0.0303), *Cxcl11* (**P = 0.0069), *Cd3e* (*P = 0.0105), and *Il17a* (*P = 0.0492). (**E**) Representative immunofluorescence images of nasal tissues at day 14 post-inoculation are shown. CD3ε (red), TCRγδ (γδ T cell) (green) and DAPI (blue). CoV2+vehicle (*n* = 3), CoV2 + ABT263 (*n* = 3). The proportion of the CD3ε^+^TCRγδ^+^ cells in total DAPI-positive cells in the olfactory mucosa is shown. Scale bar, 50 μm. *P = 0.0356. (**F**–**H**) Ten- to fourteen-week-old female wild-type (WT) and p16/p21 double knockout (DKO) mice on a C57BL/6 background were intranasally infected with a MA10, and nasal tissues were collected at the indicated time points. (**F**) Representative merged images showing SARS-CoV-2 nucleocapsid protein (CoV2-NP, red), cytokeratin 8 (CK8, a marker of sustentacular cells; green), olfactory marker protein (OMP, a marker of olfactory sensory neurons; white), and DAPI (nuclei; blue) staining in the olfactory mucosa. Scale bar, 25 μm. (**G**) Quantification of the percentage of CK8- or OMP-positive cells among CoV2-NP-positive cells at day 4 post-infection in the entire olfactory epithelium regions. *n* = 3 per group. ****P < 0.0001. (**H**) Graphs show CK8- or OMP-positive area normalised to DAPI-positive area. *n* = 3 per group. CK8/DAPI; *P = 0.0478 (WT; Mock vs. +CoV2_D4), *P = 0.0486 (WT; Mock vs. +CoV2_D14), ***P = 0.0005 (WT; +CoV2_D4 vs. +CoV2_D14), **P = 0.0027 (DKO; Mock vs. +CoV2_D4), ***P = 0.0009 (DKO; +CoV2_D4 vs. +CoV2_D14), and *ns*; *P* = 0.8127 (DKO; Mock vs. +CoV2_D14). OMP/DAPI; *P = 0.0255 (WT; Mock vs. +CoV2_D4), *P = 0.0457 (WT; +CoV2_D4 vs. +CoV2_D14), *ns*; *P* = 0.9425 (WT; Mock vs. +CoV2_D14), *P = 0.0182 (DKO; Mock vs. +CoV2_D4), *P = 0.0416 (DKO; +CoV2_D4 vs. +CoV2_D14), and *ns*; *P* = 0.8891 (DKO; Mock vs. +CoV2_D14). All data are presented as mean ± standard deviation (s.d.). Statistical significance was determined by two-way analysis of variance (ANOVA) followed by Sidak’s multiple comparison test (**B**, **G**), two-tailed unpaired *t* test (**C**–**E**) or Tukey’s multiple comparisons test (**H**). *P* values < 0.05 were considered significant. *P < 0.05, **P < 0.01, ***P < 0.001, ****P < 0.0001, ns: not statistically significant. Source data are available online for this figure.

Some, but not all, odours are unrecognisable by patients with Long COVID (Mendes Paranhos et al, 2022; Rebholz et al, 2021; Tan et al, 2022). Therefore, we performed two different olfactory tests after infecting WT and DKO mice with SARS-CoV-2. First, the olfactory avoidance test (OAT) (Kobayakawa et al, 2007; Takahashi et al, 2016), which measures olfactory function by avoidance behaviour from repellent odours, was performed on days 4 and 13 after the SARS-CoV-2 infection (Fig. EV3A). However, abnormal avoidance behaviour due to SARS-CoV-2 infection was not observed in either WT or DKO mice, at least under our experimental conditions (Fig. EV3A). This may be because the odorant used in the OAT also activates trigeminal neurons via Trpa1 (Wang et al, 2018). Consequently, we conducted an alternative test, the olfactory habituation–dishabituation test (OHDT) (Kobayakawa et al, 2007; Takahashi et al, 2016), to detect olfactory thresholds by exploratory behaviour when mice were presented with a novel odour substance (Fig. 1F,G). Notably, on day 3 after SARS-CoV-2 infection, the exploratory behaviour was reduced in both the WT and DKO mice, indicating that many were less capable of detecting eugenol (Fig. 1H). Interestingly, however, odour detection was recovered in a significant proportion of DKO mice at 12 days post-infection, in contrast to WT mice (Fig. 1H). These results correlated well with the reduced inflammatory response in the OM of DKO mice compared to WT mice (Fig. 1D), including reduced SASP factors and γδ T cell markers at 14 days post infection. Similar results were also observed when senescence-like cells were reduced by the administration of ABT263, an established senolytic drug that specifically kills senescent cells (Chang et al, 2016), in WT mice infected with SARS-CoV-2 (Figs. 2A–E and EV3B).

**Figure 3:**
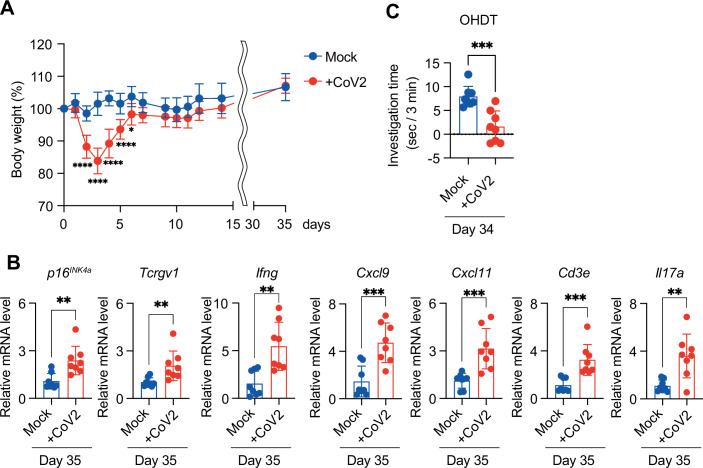
Olfactory abnormalities persist for at least 34 days post SARS-CoV-2 infection. (**A**–**C**) Ten-week-old female wild-type (WT) C57BL/6 mice were intranasally infected with a mouse-adapted strain of SARS-CoV-2 (MA10 strain; CoV2). Olfactory function was assessed by the olfactory habituation–dishabituation test (OHDT) on day 34, and the olfactory mucosa was collected on day 35 post-infection. (**A**) Body weight of infected mice was monitored until day 35 post-infection, and is shown as percentage of initial body weight (day 0) (*n* = 8 per group). *n* indicates mice (biological replicates). ****P < 0.0001, *P = 0.0112. (**B**) RT–qPCR analysis of the indicated genes in olfactory mucosa at day 35 post-infection. Gene expression levels were calculated using the ΔΔCt method and normalised to β-actin (expression level in the Mock group set as 1; *n* = 8 per group). *p16*^*INK4a*^ (**P = 0.0028), *Tcrgv1* (**P = 0.0098), *Ifng* (**P = 0.0016), *Cxcl9* (***P = 0.0007), *Cxcl11* (***P = 0.0009), *Cd3e* (***P = 0.0008), and *Il17a* (**P = 0.0022). (**C**) Olfactory function at day 34 post-infection was assessed by OHDT using eugenol as the odorant (*n* = 8 per group). ***P = 0.0005. For all graphs, error bars represent the mean ± standard deviation (s.d.). Statistical significance was determined by two-way ANOVA followed by Sidak’s multiple comparisons test (**A**), or unpaired *t* test (**B**, **C**). *P* values < 0.05 was considered significant. *P < 0.05, **P < 0.01, ***P < 0.001. Source data are available online for this figure.

The OAT utilises 2,4,5-trimethylthiazoline (TMT), a volatile compound secreted from the anal glands of predators such as foxes, which evokes a strong innate fear response in rodents (Saito et al, 2017). In contrast, eugenol—the odorant used in the OHDT—does not induce an aversive reaction and is considered neutral (Kobayakawa et al, 2007; Takahashi et al, 2016). Because of the highly aversive nature of TMT, even SARS-CoV-2-infected mice likely retained sufficient olfactory function to detect and avoid this potent repellent. By comparison, the OHDT is designed to assess the ability to detect and discriminate among neutral odorants without strong emotional responses, and may therefore be more sensitive to subtle deficits in olfactory function. This difference in sensitivity may explain why deficits were more apparent in the OHDT than in the OAT. Notably, these findings align with clinical reports suggesting that patients with Long COVID may experience selective olfactory dysfunction, in which the perception of specific odours is impaired while others remain detectable (Mendes Paranhos et al, 2022; Rebholz et al, 2021). It should also be noted that although staining for OMP (a marker of mature olfactory sensory neurons (OSNs)) (Li et al, 2004) was markedly decreased at 4 days post-infection, it had recovered to levels comparable to those in non-infected controls by 14 days post-infection (Fig. 2F–H). In addition, no substantial difference was observed between WT and DKO mice (Fig. 2F–H). Therefore, it is unlikely that the olfactory dysfunction observed at 14 days post-infection is primarily due to the impaired recovery of OSNs. Notably, body weight, gene expression, and olfactory function analyses indicated that olfactory abnormalities persisted even at 34 days post-infection (Fig. 3A–C), consistent with sustained elevation of senescence markers and SASP factors at 35 days post-infection (Fig. 3B). Together, these results suggest that the appearance of senescence-like cells indirectly induced by SARS-CoV-2 infection may be at least partly responsible for the persistent accumulation of γδ T cells in the OM, which could underlie the prolonged olfactory dysfunction seen in Long COVID patients (Finlay et al, 2022).

### γδ T cells recruited via CXCR3 signaling contribute to olfactory dysfunction

**Figure 4:**
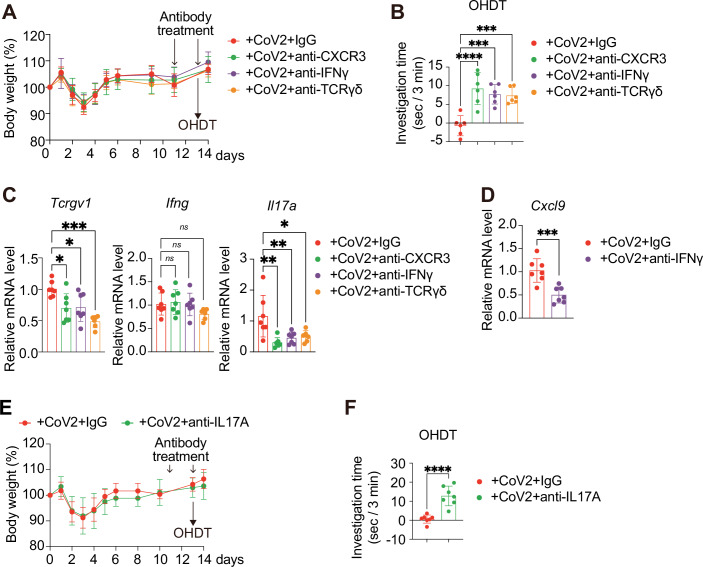
γδ T cells recruited to senescence-like cells prolong hyposmia in mice via IL-17A secretion. (**A**–**D**) Ten-week-old female WT mice were intranasally inoculated with CoV2. On days 11 and 13, neutralising antibodies were administered intraperitoneally. OHDT was performed 8–12 h after antibody administration on day 13. (**A**) Timeline of the experiments and body weight measurements. Body weight is shown as percentage of initial body weight (day 0). CoV2+IgG (*n* = 6), CoV2+anti-CXCR3 (*n* = 6), CoV2+anti-IFNγ (*n* = 6), CoV2+anti-TCRγδ (*n* = 6)). n indicates mice (biological replicates). (**B**) OHDT results using eugenol as an odorant on day 13. Each mouse was tested once. Investigation time is presented as a bar graph. CoV2+IgG (*n* = 6), CoV2+anti-CXCR3 (*n* = 6), CoV2+anti-IFNγ (*n* = 6), CoV2+anti-TCRγδ (*n* = 6). ****P < 0.0001 (+CoV2+IgG vs. +CoV2+Anti-CXCR3), ***P = 0.0006 (+CoV2+IgG vs. +CoV2+Anti-IFNγ), and ***P = 0.0009 (+CoV2+IgG vs. +CoV2+anti-TCRγδ). (**C**) RT–qPCR of the olfactory mucosa on day 14 post-inoculation was performed for *Tcrgv1*, *Ifng*, and *Il17a*. Relative mRNA expression levels were determined using the ΔΔCt method after normalisation to *β-actin*, and normalised to CoV2+IgG (set to 1). The expression level of CoV2+IgG set to 1. CoV2+IgG (*n* = 7), CoV2+anti-CXCR3 (*n* = 7), CoV2+anti-IFNγ (*n* = 7), CoV2+anti-TCRγδ (*n* = 6). *Tcrgv1*; *P = 0.0218 (+CoV2+IgG vs. +CoV2+Anti-CXCR3), *P = 0.0292 (+CoV2+IgG vs. +CoV2+Anti-IFNγ), and ***P = 0.0002 (+CoV2+IgG vs. +CoV2+anti-TCRγδ). *Ifng*; *ns*; *P* = 0.9849 (+CoV2+IgG vs. +CoV2+Anti-CXCR3), *ns*; *P* = 0.9999 (+CoV2+IgG vs. +CoV2+Anti-IFNγ), and *ns*; *P* = 0.3374 (+CoV2+IgG vs. +CoV2+anti-TCRγδ). *Il17a*; **P = 0.0017 (+CoV2+IgG vs. +CoV2+Anti-CXCR3), **P = 0.0093 (+CoV2+IgG vs. +CoV2+Anti-IFNγ), and *P = 0.0221 (+CoV2+IgG vs. +CoV2+anti-TCRγδ). (**D**) RT–qPCR of olfactory mucosa at day 14 post-inoculation was performed in mice treated with CoV2+IgG or CoV2+anti-IFNγ. The relative expression level of *Cxcl9* gene is shown. CoV2+IgG (*n* = 7), CoV2+anti-IFNγ (*n* = 7). ***P = 0.0006. (**E**) Timeline of the experiment and body weight monitoring until day 14 post-CoV2 administration. Body weight is shown as percentage of initial body weight (day 0). CoV2+IgG (*n* = 7), CoV2+anti-IL-17A (*n* = 7). (**F**) OHDT using eugenol as an odorant was performed on day 13 post-CoV2 administration. CoV2+IgG (*n* = 7), CoV2+anti-IL-17A (*n* = 7). ****P < 0.0001. All data are presented as mean ± standard deviation (s.d.). Statistical significance was determined by two-way analysis of variance (ANOVA) followed by Sidak’s multiple comparison test (**E**), two-tailed unpaired *t* test (**D**, **F**), two-way ANOVA followed by Dunnett’s multiple comparison test (**A**), or one-way ANOVA followed by Tukey’s multiple comparison test (**B**, **C**). *P* values < 0.05 were considered significant. *P < 0.05, **P < 0.01, ***P < 0.001, ****P < 0.0001, ns: not statistically significant. Source data are available online for this figure.

To further investigate the role of γδ T cells in olfactory dysfunction and clarify the mechanisms involved, we examined the effects of inhibiting cytokines and receptors that may be involved in γδ T cell recruitment and function. Remarkably, the administration of neutralising antibodies against CXCR3 (the receptor for CXCL9 and CXCL11), TCRγδ (γδ T cell receptor), and IFNγ in WT mice infected with SARS-CoV-2 significantly improved the olfactory dysfunction observed 13 days post-infection, as assessed by the OHDT (Fig. 4A,B). Furthermore, the expression level of *Tcrgv1*, a marker for γδ T cells, decreased following a treatment with any of the aforementioned antibodies (Fig. 4C). These results further support the idea that senescence-like cells promote the infiltration of γδ T cells into the OM via SASP and contribute to the persistence of olfactory abnormalities. In contrast, the administration of the aforementioned antibodies that reduce γδ T cells did not affect the *Ifng* expression levels (Fig. 4C), suggesting that the main cells in the OM that express IFNγ are not γδ T cells in this biological context. Indeed, the expression level of *Ifng* is reduced in SARS-CoV-2-infected OM when senescent cells are reduced using senolytic drugs or in DKO mice (Figs. 1D and 2D). Furthermore, consistent with previous reports that IFNγ promotes the expression of *CXCL9*, which is known to recruit γδ T cells (Ajuebor et al, 2008), the administration of a neutralising antibody against IFNγ reduced the expression level of *CXCL9* under our experimental conditions (Fig. 4D). These results indicate that the IFNγ secreted by senescence-like cells prolongs the olfactory abnormalities by recruiting γδ T cells to the OM, at least partly through CXCL9 expression. We then sought to determine how the γδ T cells accumulated in the OM cause olfactory abnormalities.

### Secretion of IL-17A by γδ T cells impairs olfactory sensory neuron function

**Figure EV4:**
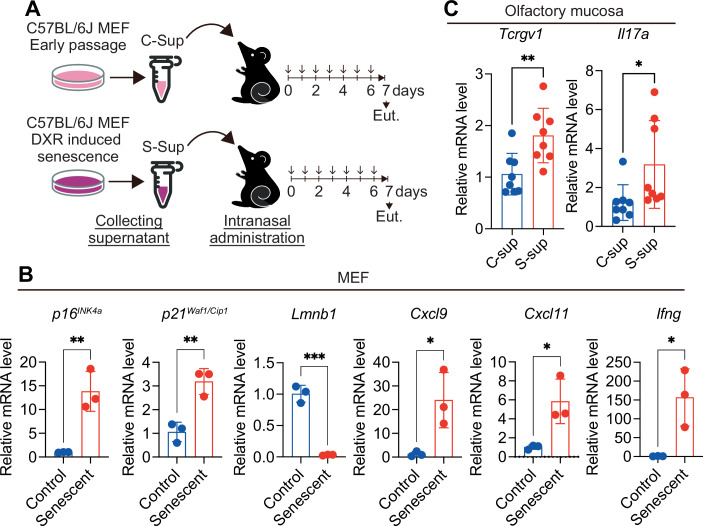
Intranasal administration of senescent cell supernatant recruits γδ T cells to the olfactory mucosa in mice. (**A**) Experimental design. Early-passage mouse embryonic fibroblasts (MEFs) derived from wild-type C57BL/6 mice were rendered senescent by treatment with 100 ng/mL doxorubicin (DXR) for 9 days. Afterward, the culture medium was replaced, and senescent MEFs were seeded at 6 × 10⁵ cells per 35-mm dish and incubated for an additional 3 days. Supernatants were then collected (senescent MEF supernatant: S-sup). Control early-passage MEFs (≤6 passages) were seeded at the same density and incubated under identical conditions, and their supernatants were collected (control MEF supernatant: C-sup). Each supernatant (20 μL) was intranasally administered to C57BL/6 mice under anesthesia once daily for 7 consecutive days. Olfactory mucosa was subsequently collected for RT–qPCR analysis. Euthanasia (Eut.) (**B**) RT–qPCR analysis of gene expression in MEFs used for supernatant collection. The expression of senescence markers (*p16*^*INK4a*^, *p21*^*Waf1/Cip1*^, and *Lmnb1*) and SASP-related genes (*Cxcl9, Cxcl11*, and *Ifng*) was quantified using the ΔΔCt method and normalized to 18 s rRNA. qPCRs were performed for 40 cycles. ‘Undetermined’ data points were assigned a Ct of ‘40’ to enable calculation of fold change. Control, *n* = 3; Senescent, *n* = 3. n indicates mice (biological replicates). *p16*^*INK4a*^ (**P = 0.0062), *p21*^*Waf1/Cip1*^ (**P = 0.0057), *Lmnb1* (***P = 0.0002), *Cxcl9* (*P = 0.0281), *Cxcl11* (*P = 0.0238), and *Ifng* (*P = 0.0235). (**C**) RT–qPCR analysis of *Tcrgv1* and *Il17a* expression in the olfactory mucosa of mice intranasally administered MEF supernatants. *n* = 8. Expression levels were calculated using the ΔΔCt method and normalized to β-actin (C-sup set to 1). *Tcrgv1* (**P = 0.0066) and *Il17a* (*P = 0.0392). All data are presented as mean ± standard deviation (s.d.). Statistical significance was determined using an unpaired *t* test. *P* values < 0.05 were considered significant. *P < 0.05, **P < 0.01, ***P < 0.001, ****P < 0.0001.

**Figure 5:**
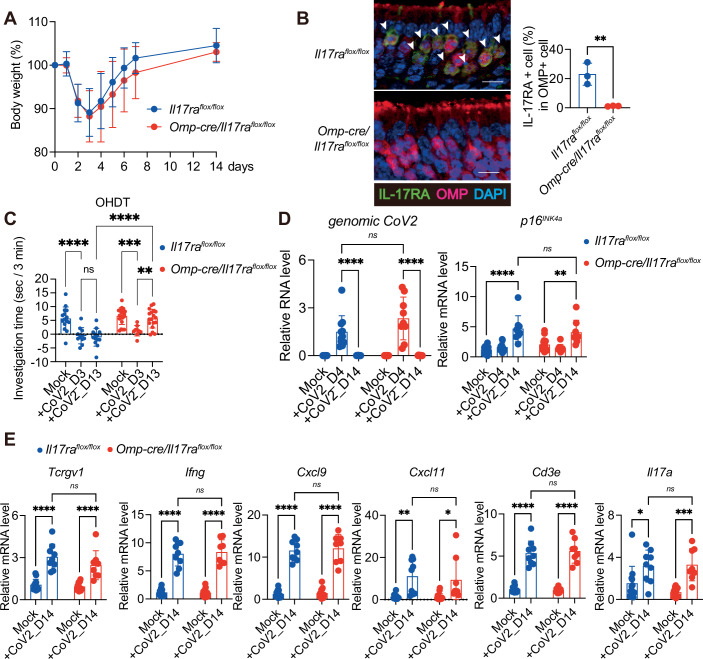
SARS-CoV-2-induced hyposmia is attenuated in mice lacking IL-17RA specifically in olfactory sensory neurons. Ten- to fourteen-week-old female *Il17ra*^*flox/flox*^ or *Omp-cre/Il17ra*^*flox/flox*^ (olfactory sensory neuron–specific IL-17 receptor A knockout) mice on a C57BL/6 background were intranasally infected with a mouse-adapted strain of SARS-CoV-2 (MA10; CoV2). Olfactory function was assessed using the olfactory habituation–dishabituation test (OHDT) on day 3 or 13, and total RNA was extracted from the olfactory mucosa on day 14. (**A**) Body weight was monitored through day 14 post-infection, and is shown as percentage of initial body weight (day 0). (*Il17ra*^*flox/flox*^: *n* = 12; *Omp-cre/Il17ra*^*flox/flox*^: *n* = 12). n indicates mice (biological replicates). (**B**) Representative immunofluorescence images showing IL-17RA (green), OMP (red), and DAPI (blue). Arrowheads indicate IL-17RA+ cells. Scale bar, 10 μm. Quantification shows the percentage of IL-17RA-positive cells among OMP-positive cells in the dorsal lateral turbinate area of olfactory epithelium (defined as the region above the horizontal basal cell layer; 500 μm × 700 μm) (*n* = 3). **P = 0.0074. (**C**) OHDT was performed on day 3 or 13 using eugenol as a novel odorant. To avoid confounding owing to learning, each mouse was used only once in the test. Investigation time is presented as a bar graph. *Il17ra*^*flox/flox*^: Mock (*n* = 15), day 3 (*n* = 11), day 13 (*n* = 13); OMP- *Omp-cre/Il17ra*^*flox/flox*^: Mock (*n* = 20), day 3 (*n* = 9), day 13 (*n* = 18). ****P < 0.0001, ***P = 0.0006, **P = 0.0078, and *ns*; *P* = 0.9976. (**D**, **E**) RT–qPCR analysis of olfactory mucosa at days 4 and 14 post-infection (**D**) or day 14 (**E**). Relative mRNA expression level was determined using the ΔΔCt method after normalization to *β-actin*. The expression level in the *Il17ra*^*flox/flox*^ +Mock group was set to 1. Relative expression of *genomic CoV2* normalised to *β-actin* is shown. *Il17ra*^*flox/flox*^: Mock (*n* = 12), day 4 (*n* = 11), day 14 (*n* = 9); *Omp-cre/Il17ra*^*flox/flox*^: Mock (*n* = 16), day 4 (*n* = 9), day 14 (*n* = 8). *Genomic CoV2* (****P < 0.0001, *ns*; *P* = 0.0561), *p16*^*INK4a*^ (****P < 0.0001, **P = 0.0059, *ns*; *P* = 0.9163), *Tcrgv1* (****P < 0.0001, *ns*; *P* = 0.3493), *Ifng* (****P < 0.0001, *ns*; *P* = 0.9978), *Cxcl9* (****P < 0.0001, *ns*; *P* = 0.9948), *Cxcl11* (**P = 0.0023, *P = 0.0141, *ns*; *P* = 0.9898), *Cd3e* (****P < 0.0001, *ns*; *P* = 0.9942), and *Il17a* (*P = 0.0323, ***P = 0.0002, *ns*; *P* > 0.9999). All data are presented as mean ± standard deviation (s.d.). Statistical significance was determined by two-way analysis of variance (ANOVA) followed by Sidak’s multiple comparison test (**A**, **C**, **E**), two-tailed unpaired *t* test (**B**), or two-way ANOVA followed by Tukey’s multiple comparison test (**D**). *P* values < 0.05 were considered significant. *P < 0.05, ** *P* < 0.01, *** *P* < 0.001, **** *P* < 0.0001, ns: not significant. Source data are available online for this figure.

**Figure EV5:**
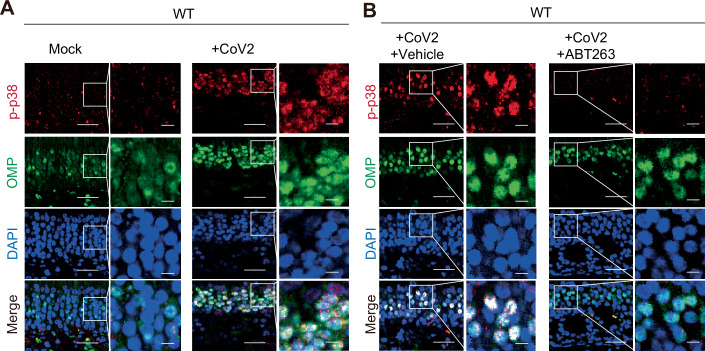
Senolysis suppresses sustained p38-MAPK activation in olfactory sensory neurons of SARS-CoV-2-infected mice. Ten- to fourteen-week-old female wild-type (WT) mice were intranasally inoculated with SARS-CoV-2 (MA10; CoV2) and treated with ABT263 or vehicle. The administration schedule for ABT263 is shown in Fig. 2A. All mice were euthanized on day 14 post-infection, and the olfactory mucosa was subjected to immunofluorescence analysis. Representative images showing Phospho-p38 (red), OMP (green), and 4′,6-diamidino-2-phenylindole (DAPI; blue). Areas enclosed by white boxes in each image are shown at higher magnification on the right. Scale bars, 25 μm (low magnification) and 5 μm (high magnification). (**A**) Mock (*n* = 3); +CoV2 (*n* = 4). (**B**)  + CoV2 + Vehicle (*n* = 5); +CoV2 + ABT263 (*n* = 5). *n* indicates mice (biological replicates).

IL-17A is one of the cytokines secreted by γδ T cells (Douglas et al, 2023) (Fig. EV3C) and reportedly disrupts neuronal function in the central nervous system (Alves de Lima et al, 2020; Brigas et al, 2021). Notably, the expression level of *Il17a* after SARS-CoV-2 infection was reduced not only by administering the aforementioned neutralising antibody that inhibits γδ T accumulation (Fig. 4C), but also by genetic or pharmacological inhibition of senescence-like cell accumulation (Figs. 1D and 2D). Conversely, intranasal administration of conditioned medium from senescent cells induces γδ T cell accumulation and *Il17a* expression in the nasal mucosa of young mice (Fig. EV4). Furthermore, the administration of IL-17A neutralising antibodies resulted in the recovery of olfactory dysfunction, as assessed by the OHDT, in WT mice 13 days after SARS-CoV-2 infection (Fig. 4E,F). However, since neutralising antibodies and senolytic drugs act systemically, it is difficult to conclude that these observations reflect an OSNs-specific effect. To address this limitation, we generated mice lacking IL-17 receptor A specifically in OSNs, by crossing *Omp-cre* mice (Li et al, 2004) with *Il17ra*^*flox/flox*^ mice (Kumar, Monin et al, 2016) (Fig. 5A,B). In these OMP-specific *Il17ra* knockout mice, the prolonged olfactory dysfunction was markedly alleviated, as assessed by the OHDT (Fig. 5C). Importantly, the expression levels of senescence markers (*p16*^*INK4a*^), SASP factor genes (*Cxcl9, Cxcl11, Ifng*), and γδ T cell markers (*Tcrgv1, Cd3e, Il17a*) were comparably elevated in both *Il17ra*^*flox/flox*^ and *Omp-cre/Il17ra*^*flox/flox*^ mice following SARS-CoV-2 infection (Fig. 5D,E), indicating that the deletion of IL-17 receptor A in OSNs does not affect the upstream senescence–γδ T cell axis but specifically blocks the downstream effect of IL-17A on olfactory neurons. Collectively, these findings indicate that the senescence-like cells that emerge upon SARS-CoV-2 infection persist in the OM even after the virus has disappeared, and their secretion of SASP factors recruits γδ T cells, which in turn secrete IL-17A that sustains olfactory abnormalities. It is also worth noting that stimulation of the IL-17 receptor A reportedly impairs neuronal function via aberrant activation of p38 MAPK (Di Filippo et al, 2021). Consistent with this, in WT mice, increased phosphorylation of p38 MAPK, a marker of its activation, was observed in OSNs 14 days after SARS-CoV-2 infection, but was reduced by ABT263 treatment (Fig. EV5). Taken together, our findings indicate that the persistent presence of senescence-like cells in the OM after SARS-CoV-2 infection leads to the long-term accumulation of γδ T cells in the OM via the secretion of SASP factors. This may cause the excessive activation of the p38 pathway in OSNs by the IL-17A released from γδ T cells, contributing at least in part to the protracted olfactory abnormalities in Long COVID.

It should be noted, however, that unlike the mouse model described here, both the symptoms and incidence of Long COVID vary widely in humans, with only a subset of patients experiencing persistent hyposmia (Tan et al, 2022). Although the underlying causes of this heterogeneity remain unclear, factors such as genetic background, lifestyle, commensal microbiota, and viral load may contribute. In contrast, these variables are largely uniform in laboratory mouse experiments, which may explain the differing frequencies of olfactory abnormalities observed following SARS-CoV-2 infection in humans and mice. In addition, a key limitation of the present study is that the translational relevance of these mouse-derived findings to patients with Long COVID remains to be established. Although publicly available human single-cell transcriptome datasets from Long COVID patients exist, these datasets do not contain sufficient numbers of the key cell types of interest—namely fibroblasts, γδ T cells, and mature olfactory sensory neurons—to adequately test whether the senescence-associated mechanisms observed in our mouse model are also present in humans. This limitation likely reflects the technical and ethical challenges of obtaining deep olfactory epithelial samples from well-characterized Long COVID patients. Nonetheless, we anticipate that our findings may inform future investigations into the pathogenesis of post COVID-19 olfactory dysfunction.

## Methods

**Table:** Reagents and tools table

Reagent/resource	Reference or source	Identifier or catalog number
**Experimental models**		
Mouse model		“Methods”
Normal human lung diploid fibroblasts (HDFs; TIG-3)	Japanese Cancer Research Resources Bank (JCRB)	JCRB0506
VeroE6/TMPRSS2 cells	Japanese Cancer Research Resources Bank (JCRB)	JCRB1819
**Antibodies**		
p16^INK4a^	Santa Cruz	sc-56330
SARS-CoV-2 nucleocapsid protein	Sino Biological	40143-R001
p16^INK4a^	Abcam	ab211542
CXCL9	R&D Systems	AF-492
Ki67	Thermo Fisher Scientific	14-5698-82
SARS-CoV-2 spike protein	GeneTex	GTX632604
TCRγδ	Santa Cruz	sc-19608
TCRγδ	LSBio	LS-B5684
phospho-p38 MAPK	Cell Signaling	4511
OMP (olfactory marker protein)	Wako	019-22291
cytokeratin 8	Abcam	ab53280
IL-17RA	Abcam	ab180904
CD3 epsilon	Abcam	ab16669
IL-17A	Invitrogen	PA5-114455
Armenian hamster IgG isotype control	Bio X Cell	BE0091
Anti-mouse CXCR3	Bio X Cell	BE0249
Anti-mouse IFNγ	Bio X Cell	BE0312
Anti-mouse TCRγ/δ	Bio X Cell	BE0070
Mouse IgG1 isotype control	Bio X Cell	BE0083
Anti-mouse IL-17A	Bio X Cell	BE0173
Donkey anti-Mouse IgG Alexa Fluor 488	Thermo Fisher	A-21202
Donkey anti-Rabbit IgG Alexa Fluor 555	Thermo Fisher	A-31572
Donkey anti-Goat IgG Alexa Fluor 488	Thermo Fisher	A-11055
Goat Anti-Armenian hamster Alexa Fluor 568	abcam	ab175716
Donkey anti-Rabbit IgG Alexa Fluor Plus 488	Thermo Fisher	A-32790
Donkey anti-Goat IgG Alexa Fluor Plus 555	Thermo Fisher	A-32816
ImmPRESS Polymer Anti-Rabbit IgG	Vector	MP-7401
ImmPRESS Polymer Anti-Goat IgG	Vector	MP-7405
Peroxidase-conjugated AffiniPure Goat Anti-Armenian Hamster IgG	ImmunoResearch Laboratories	127-035-160
**Oligonucleotides and other sequence-based reagents**		
human *TNFα* 5’-GCCCCCAGAGGGAAGAGTTCCCCA-3’,	Tsuji et al, 2022	
human *TNFα* 5’-GCT TGAGGGTTTGCTACAACATGGGC-3’,	Tsuji et al, 2022	
human *IL-6* 5’- CCAGGAGCCCAGCTATGAAC-3’,	Tsuji et al, 2022	
human *IL-6* 5’-CCCAGGGAGAAGGCAACTG-3’,	Tsuji et al, 2022	
human *IFNβ* 5’- AAACTCATGAGCAGTCTGCA -3’,	Tsuji et al, 2022	
human *IFNβ* 5’- AGGAGATCTTCAGTTTCGGAGG-3’,	Tsuji et al, 2022	
human *IL-1β* 5’- CTGTCCTGCGTGTTGAAAGA-3’,	Tsuji et al, 2022	
human *IL-1β* 5’- TTGGGTAATTTTTGGGATCTACA-3’,	Tsuji et al, 2022	
human *IL-8* 5’- AAGGAAAACTGGGTGCAGAG-3’,	Tsuji et al, 2022	
human *IL-8* 5’- ATTGCATCTGGCAACCCTAC-3’,	Tsuji et al, 2022	
human *GAPDH* 5’- CAACTACATGGTTTACATGTTC-3’,	Tsuji et al, 2022	
human *GAPDH* 5’- GCCAGTGGACTCCACGAC-3’,	Tsuji et al, 2022	
mouse *β-actin* 5’- GATGACCCAGATCATGTTTGA-3’,	Kawamoto et al, 2023	
mouse *β-actin* 5’- GGAGAGCATAGCCCTCGTAG-3’,	Kawamoto et al, 2023	
mouse *p16*^*INK4a*^ 5’- GAACTCTTTCGGTCGTACCC-3’,	Kawamoto et al, 2023	
mouse *p16*^*INK4a*^ 5’- CGAATCTGCACCGTAGTTGA-3’,	Kawamoto et al, 2023	
mouse *Ifng* 5’- ATGAACGCTACACACTGCATC-3’,	Tsuji et al, 2022	
mouse *Ifng* 5’- CCATCCTTTTGCCAGTTCCTC-3’,	Tsuji et al, 2022	
mouse *Cxcl9* 5’- CCTAGTGATAAGGAATGCACGATG-3’,	Tsuji et al, 2022	
mouse *Cxcl9* 5’- CTAGGCAGGTTTGATCTCCGTTC-3’,	Tsuji et al, 2022	
SARS-CoV-2 N 5’- TTACAAACATTGGCCGCAAA-3’,	Tsuji et al, 2022	
SARS-CoV-2 N 5’- GCGCGACATTCCGAAGAA-3’,	Tsuji et al, 2022	
SARS-CoV-2 subgenomic N 5’- CCAGGTAACAAACCAACCAACTTTCG-3’,	Tsuji et al, 2022	
SARS-CoV-2 subgenomic N 5’- GGTTACTGCCAGTTGAATCTGAGG-3’,	Tsuji et al, 2022	
mouse *Cxcl11* 5’-GCCATAGCCCTGGCTGCGAT-3’,	This study	
mouse *Cxcl11* 5’-CATCCCGGGGCCGATGCAAA-3’,	This study	
mouse *Il17a* 5’-GCTCCAGAAGGCCCTCAGA-3’,	This study	
mouse *Il17a* 5’-CTTTCCCTCCGCATTGACA-3’,	This study	
mouse *Tcrgv1* 5’-ACGACCCTTAGGAGGGAAGA-3’,	This study	
mouse *Tcrgv1* 5’- CTGCACAGTAGTAGGTGGCT-3’,	This study	
mouse *Cd3e* 5’-AACACTTTCTGGGGCATCCT-3’,	This study	
mouse *Cd3e* 5’-ATGTTCTCGGCATCGTCCT-3’,	This study	
mouse *p21*^*Waf1/Cip1/Sdi1*^ 5’-TGTCTTGCACTCTGGTGTCT-3’,	This study	
mouse *p21*^*Waf1/Cip1/Sdi1*^ 5’-TGAGGGCTAAGGCCGAAGAT-3’,	This study	
mouse *Lmnb1* 5’-GAGTATGAGGCGGCACTAAAC-3’,	This study	
mouse *Lmnb1* 5’-CATCTGCTAACTGCTTTTTGGC-3’,	This study	
mouse *18S rRNA* 5’-CTCAACACGGGAAACCTCAC-3’,	This study	
mouse *18S rRNA* 5’- CGCTCCACCAACTAAGAACG-3’	This study	
**Chemicals, enzymes and other reagents**		
**Software**		
Prism v10.4.2	https://www.graphpad.com/features	
**Other**		

### Animal experiments

All of the animal experiments were approved by the Animal Research Committee of the Research Institute for Microbial Diseases (RIMD), The University of Osaka (approval number: R07-05-0, R02-08-0). Mice were euthanized using carbon dioxide, and all efforts were made to minimize suffering. WT C57BL/6 mice were purchased from CLEA Japan. p16/p21-DKO mice were described previously (Kawamoto et al, 2023). *p16*^*flox/flox*^ mice (Liu et al, 2011) were kindly provided by Prof. Dr. Norman E Sharpless. *Pdgfra-creER*^*TM*^ (B6N.Cg-Tg(*Pdgfra-creER*^*TM*^) 467Dbe/J) mice (Strain #:018280) were described previously (Kang et al, 2010). *Omp-cre* (B6;129P2(Cg)-Omp<tm4(cre)Mom > /MomTyagRbrc) mice were provided by the RIKEN BRC (BRC No. RBRC02138) through the National Bio-Resource Project of the MEXT/AMED, Japan. *Il17ra*^*flox/flox*^ (B6.Cg-Il17ratm2.1Koll/J) mice (Strain #:031000) were provided by the Jackson Laboratory. All mice were bred and maintained in a specific pathogen-free (SPF) facility at the Research Institute for Microbial Diseases (RIMD), The University of Osaka, under controlled conditions (23 ± 2 °C, 55 ± 15% humidity, and a 12-h light/dark cycle) and fed a normal diet (CE-2, CLEA Japan; sterilized by 20 kGy gamma irradiation). Prior to SARS-CoV-2 infection, mice were transferred to the ABSL-3 facility at RIMD at least 5 days in advance. Female mice aged 10 to 14 weeks were used for infection experiments. Mice were randomly assigned to experimental groups prior to virus infection and drug administration. Mice were intranasally inoculated with SARS-CoV-2 MA10 (1.0 × 10⁶ PFU in 50–80 μL) under isoflurane anesthesia. Following recovery from anaesthesia, mice received oral administration of ABT263 (Selleck, S1001), dissolved in a mixture of 10% ethanol (Nacalai, 14712-05), 30% polyethylene glycol 400 (PEG-400; Nacalai, 28215-95), and 60% Phosal 50 PG (H. Holstein). The dose of ABT263 was 100 mg/kg per administration. For neutralising antibody experiments, SARS-CoV-2 (MA10)-infected mice were intraperitoneally injected with 200 μg (Armenian hamster IgG, anti-CXCR3, anti-IFNγ, or anti-TCRγ/δ) or 250 μg (mouse IgG1 or anti-IL-17A) of neutralising antibody diluted in 150 μL sterile PBS on days 11 and 13 post-infection. All antibodies were purchased from Bio X Cell: Armenian hamster IgG isotype control (BE0091, lot 842222M1), anti-mouse CXCR3 (BE0249, lot 782621N1), anti-mouse IFNγ (BE0312, lot 809722F1), anti-mouse TCRγ/δ (BE0070, lot 762421J2), mouse IgG1 isotype control (BE0083, lot 785121O1), and anti-mouse IL-17A (BE0173, lot 808922A1).

For the intranasal administration experiment using conditioned medium derived from mouse embryonic fibroblasts (MEFs), mice were anesthetized with a combination of medetomidine, midazolam, and butorphanol. A total of 20 μL of conditioned medium from either senescent or control (early-passage) cells was administered intranasally once daily for 7 consecutive days. Twenty-four hours after the final administration, the olfactory epithelium was harvested, and total RNA was extracted for RT–qPCR analysis. The preparation of conditioned medium is described in the “Cell culture” section.

### Biosafety

All experiments involving SARS-CoV-2 were performed in a biosafety level 3 (BSL-3) facility at the RIMD, The University of Osaka (Osaka, Japan), in accordance with institutional and governmental biosafety regulations. All experimental protocols were approved by the Institutional Animal Care and Use Committee of the RIMD, The University of Osaka (approval number: 02-08-0). The production of recombinant SARS-CoV-2 used in this study was approved by the Institutional Biosafety Committee for recombinant DNA experiments of The University of Osaka (approval number: 4625).

### Cell culture

Normal human lung diploid fibroblasts (HDFs; TIG-3, JCRB0506), Mouse embryonic fibroblasts (MEFs), and VeroE6/TMPRSS2 cells (JCRB1819) were cultured in Dulbecco’s Modified Eagle Medium (DMEM) supplemented with 10% foetal bovine serum (FBS; MP Biomedicals, 2917354H) and 100 U/mL penicillin–streptomycin (Sigma, P4333). ACE2-expressing HDFs (ACE2-HDFs) were generated via retroviral transduction as described previously (Tsuji et al, 2022). Briefly, cells were infected with retroviruses encoding human ACE2 (pMarX-hygro) and selected with 50 μg/mL hygromycin for 24 h. MEFs derived from wild-type C57BL/6 mice were rendered senescent by treatment with 100 ng/mL doxorubicin (DXR) for 9 days. After senescence induction, the cells were seeded at a density of 6 × 10⁵ cells per 35-mm dish and cultured for an additional 3 days. The resulting culture supernatants were collected and referred to as senescent MEF supernatant (S-sup). Control MEFs (≤6 passages) were seeded at the same density and cultured under identical conditions, and their supernatants were collected as control MEF supernatant (C-sup). Cell debris was removed by centrifugation at 300×*g* for 10 min. All cell lines were routinely tested for mycoplasma contamination and confirmed to be negative.

### Virus preparation

A mouse-adapted strain of SARS-CoV-2 (MA10), generated by reverse genetics described as CIPER (Torii et al, 2021), was propagated in Vero/TMPRSS2 cells for 48 h, and viral titres were determined using the TCID50 assay.

### In vitro SARS-CoV-2 infection experiments

ACE2-HDFs were seeded at 2 × 10⁴ cells per well in 6-well plates and cultured for 24 h at 37 °C in 5% CO₂. Prior to infection, cells were washed twice with PBS, and the medium was replaced with DMEM supplemented with 2% FBS. Cells were then infected with SARS-CoV-2 MA10 at a multiplicity of infection (MOI) of 0.1. At 24 h post-infection, cells were washed twice with PBS and the medium was replaced with standard culture medium.

### Immunocytochemistry

Cells were fixed with 4% paraformaldehyde (Nacalai, 09154-85) for 25 min at room temperature. Immunocytochemistry was performed using primary antibodies against SARS-CoV-2 nucleocapsid protein (Sino Biological, 40143-R001; 1:1000) and human p16^INK4a^ (Santa Cruz, sc-56330; 1:200). Secondary antibodies used were donkey anti-mouse IgG Alexa Fluor 488 (Thermo Fisher, A-21202; 1:1000) and donkey anti-rabbit IgG Alexa Fluor 555 (Thermo Fisher, A-31572; 1:1000). Nuclei were counterstained with 4’,6-diamidino-2-phenylindole (DAPI; Dojindo, 340-07971; 1:2000).

### Immunohistochemistry

Following euthanasia by isoflurane overdose, the head was surgically removed and the overlying skin and lower jaw were carefully dissected. After washing with PBS, tissues were promptly fixed in either Bouin’s solution (Muto Pure Chemicals, 33142) for 2 h at 4 °C, or 4% paraformaldehyde (FUJIFILM, 162-16065) for 24 h at 4 °C. Following three PBS washes, tissues were decalcified in 10% (w/v) EDTA (Muto Pure Chemicals, 20251) at 4 °C for 7 days on a shaker at 70 rpm. After additional PBS washes, the olfactory mucosa was excised, transferred to 70% ethanol, and embedded in paraffin. Tissue sections were cut at a thickness of 5 μm using a rotary microtome, mounted on MAS-coated glass slides (Matsunami), and air-dried overnight at 37 °C. Slides were deparaffinised with PathoClean (Fujifilm Wako), rehydrated through a graded ethanol series, and subjected to antigen retrieval in citrate buffer (pH 6.0) via microwave heating for 15 min.

Sections fixed with Bouin’s solution were used for p16^INK4a^ staining. After antigen retrieval, sections were permeabilised with 0.1% Triton X-100 in PBS for 10 min at room temperature, followed by quenching of endogenous peroxidase activity with BLOXALL (Vector, SP-6000) for 10 min. Blocking was carried out using 2.5% normal horse serum for 30 min at room temperature. Sections were incubated overnight at 4 °C with the following primary antibodies: mouse anti-p16^INK4a^ (Abcam, ab211542; 1:200) (Kawamoto et al, 2023), anti-CXCL9 (R&D Systems, AF-492; 1:20), and anti-Ki67 (Thermo Fisher Scientific, 14-5698-82; 1:200). The following secondary antibodies were then applied for 30 min at room temperature: ImmPRESS Polymer Anti-Goat IgG (Vector, MP-7405) and ImmPRESS Polymer Anti-Rabbit IgG (Vector, MP-7401). Signal amplification was performed using either the TSA Cyanine 5 System (AKOYA Biosciences, NEL705A001KT) or the TSA TMR System (AKOYA Biosciences, NEL702001KT), according to the manufacturer’s instructions. Nuclei were counterstained with DAPI (Dojindo, 340-07971; 1:1000) for 1 h, and slides were mounted with Fluoromount-G (SouthernBiotech, 0100-01). For dual staining of p16^INK4a^ and SARS-CoV-2 spike protein, sections were first stained for p16^INK4a^ as described above. Subsequent staining for the SARS-CoV-2 spike protein was performed using a primary antibody (GeneTex, GTX632604; 1:1000) in combination with the VECTOR M.O.M. Immunodetection Kit (Vector, BMK-2202), following the manufacturer’s instructions. Donkey anti-mouse IgG Alexa Fluor 488 (Thermo Fisher, A-21202; 1:1000) was used as the secondary antibody.

Immunohistochemical analysis of 4% PFA-fixed sections was carried out using the following primary antibodies: anti-TCRγδ (Santa Cruz, sc-19608; 1:50), anti-phospho-p38 MAPK (Cell Signalling Technology, 4511; 1:500), anti-OMP (Wako, 019-22291; 1:1000), anti-cytokeratin 8 (Abcam, ab53280; 1:250), anti-IL-17RA (Abcam, ab180904; 1:50), anti-SARS-CoV-2 nucleocapsid protein (Sino Biological, 40143-R001; 1:1000), and anti-IL-17A (Invitrogen, PA5-114455; 1:1000). The corresponding secondary antibodies included: donkey anti-rabbit IgG Alexa Fluor Plus 488 (Thermo Fisher, A-32790; 1:1000), donkey anti-rabbit IgG Alexa Fluor 555 (Thermo Fisher, A-31572; 1:1000), goat anti-Armenian hamster IgG Alexa Fluor 568 (Abcam, ab175716; 1:1000), donkey anti-goat IgG Alexa Fluor 488 (Thermo Fisher, A-11055; 1:1000), and donkey anti-goat IgG Alexa Fluor Plus 555 (Thermo Fisher, A-32816; 1:1000). Immunostaining procedures were performed as described above. Signal amplification for IL-17RA, IL-17A, and SARS-CoV-2 nucleocapsid protein was achieved using the TSA Cyanine 5 or TMR Systems (AKOYA Biosciences).

For frozen sections, after decalcification, samples were washed three times in PBS, soaked overnight in 30% sucrose in PBS, embedded in Tissue-Tek cryomolds (4557; Sakura Finetek) using O.C.T. compound (45833; Sakura Finetek), and frozen. Sections were cut at a thickness of 10 µm using a cryostat (Leica Biosystems). Immunohistochemical analysis of frozen sections was performed using the following primary antibodies: anti-TCRγδ (LSBio, LS-B5684; 1:50) and anti-CD3ε (Abcam, ab16669; 1:150). For the first staining round (TCRγδ), endogenous peroxidase activity was quenched with BLOXALL (Vector, SP 6000) for 10 min, followed by blocking with 2.5% normal goat serum for 30 min at room temperature. Sections were incubated overnight at 4 °C with anti-TCRγδ, followed by incubation with peroxidase-conjugated AffiniPure Goat Anti-Armenian Hamster IgG (ImmunoResearch Laboratories, 127-035-160; 1:1000) for 30 min at room temperature. Signal was developed using the TSA TMR system (AKOYA Biosciences, NEL702001KT) according to the manufacturer’s instructions. After two washes in PBS, sections underwent a second round of antigen retrieval in citrate buffer (pH 6.0) by heating for 15 min. Endogenous peroxidase activity was again quenched with BLOXALL for 10 min, followed by blocking with 2.5% normal horse serum for 30 min at room temperature. Sections were incubated for 3 h at room temperature with anti-CD3ε, followed by ImmPRESS Polymer Anti-Rabbit IgG (Vector, MP-7401) for 30 min at room temperature. Signal was amplified using the TSA Cyanine 5 system (AKOYA Biosciences, NEL705A001KT). Nuclei were counterstained with DAPI (Dojindo, 340-07971; 1:1000) for 30 min, and slides were mounted with Fluoromount-G (SouthernBiotech, 0100-01).

Multiplex immunohistochemistry for SARS-CoV-2 nucleocapsid protein, cytokeratin 8, and OMP was performed as follows: after signal amplification of the nucleocapsid protein using the TSA Cyanine 5 System, sections underwent a second round of antigen retrieval in citrate buffer (pH 6.0) via microwave heating for 10 min. Sections were then blocked with 5% normal donkey serum and incubated overnight at 4 °C with antibodies against cytokeratin 8 and OMP. Secondary antibodies used included donkey anti-rabbit IgG Alexa Fluor Plus 488 and donkey anti-goat IgG Alexa Fluor Plus 555. Sections were mounted using Fluoromount-G.

Fluorescence images were acquired using a BZ-X710 or BZ-X800 All-in-One Fluorescence Microscope (Keyence) equipped with either a Plan Apochromat 20×/0.75 or 40×/0.95 objective lens under standardised exposure conditions.

The BZ-X filters used included DAPI (OP-87762), GFP (OP-87763), TRITC (OP-87764), and Cy5 (OP-87766). Images were acquired under constant exposure conditions across all samples, with consistent gain and offset settings (Montero Llopis et al, 2021). For multiplex staining, sequential image acquisition was performed to avoid spectral bleed-through. To quantify SARS-CoV-2 infection and OMP- or cytokeratin 8 (CK8)-positive areas, the entire olfactory epithelium was imaged using the image stitching function of the BZ-X Analyzer software (Keyence). The olfactory epithelial layer was defined as the region above the horizontal basal cell layer and was manually extracted from each image using Photoshop (Adobe). Identical cropping regions were applied to all fluorescence channels of the same sample to ensure consistency. Processed images were imported into ImageJ (NIH), binarised using a fixed threshold, and the area of positive signal for each marker was automatically quantified.

For multicolor immunofluorescence quantification, merged fluorescence images were analysed using the Hybrid Cell Count module of the BZ-X Analyzer software (Keyence). A threshold for the marker of interest was first determined—typically using the automatic setting—based on signal distribution across all samples. The positive area for that marker was then quantified. To assess co-localization with additional signals, thresholds were individually set for each additional marker, and positive areas were identified within the previously defined marker-positive regions. Finally, DAPI-positive nuclei within these co-localized regions were counted to determine the number of double-positive cells. Quantification was performed using either stitched images covering the entire half of the olfactory epithelium or non-merged images from at least three independent fields within the dorsolateral turbinate area. Fluorescence images of TCRγδ and CD3ε staining were acquired using a Leica Mica confocal microscope (Leica Microsystems). Images were captured under constant exposure conditions across all samples, with identical gain and offset settings. Image files were imported into ImageJ (NIH), binarized using a fixed threshold, and TCRγδ⁺CD3ε⁺DAPI⁺ triple-positive cells were counted. Quantification was performed using unmerged images from at least five independent microscopic fields within the dorsolateral turbinate region.

### RNA isolation and quantitative real-time PCR

For RNA extraction from the olfactory mucosa, mice were euthanised by isoflurane overdose. The head was surgically removed, and the overlying skin and lower jaw were carefully dissected. A longitudinal incision was made along the sagittal midline of the cerebrum, extending from the occipital region to the nasal tip, using surgical scissors. The olfactory mucosa, visually identifiable beneath the olfactory bulbs, was carefully dissected using fine forceps, taking care to avoid excessive scraping. Following dissection, tissues were immediately immersed in TRIzol Reagent (Thermo Fisher Scientific, 15596026) containing zirconia beads and homogenised using a bead crusher (Bertin Technologies, Precellys Evolution) at 6,500 rpm for 30 s, repeated three times with 10-second intervals. Total RNA was extracted according to the manufacturer’s instructions. For reverse transcription, 1.0 μg of total RNA was used with the PrimeScript RT Reagent Kit (Takara Bio Inc., PR047A) to synthesise complementary DNA (cDNA). RT–qPCR was performed using the Thermal Cycler Dice® Real Time System III (Takara Bio) or QuantStudio 1 (Thermo Fisher Scientific) with TB Green® Premix Ex Taq™ II (Takara Bio, RR820A). Each 25 μL reaction mixture contained 12.5 μL of master mix, 1 μL each of 10 μM forward and reverse primers, 15–52.5 ng of cDNA template, and RNase-free water. Relative mRNA levels were calculated using the ΔΔCt method, normalised to β-actin or 18 s rRNA for mouse samples or GAPDH for human cell samples, and expressed relative to mock-treated controls (set to 1.0). Relative RNA levels were calculated as 2^−ΔCt^. The primer sequences used in this study are listed in the Reagents & Tools Table.

### Olfactory avoidance test

The olfactory avoidance test (OAT) (Kobayakawa et al, 2007; Takahashi et al, 2016) was adapted for use under BSL-3 containment conditions. Each mouse was tested only once to avoid potential confounding effects of learning. The test cage (TECNIPLAST, 1245ISO00DPBOX; 365 × 207 × 140 mm) was equally divided into two compartments (1:1), allowing free movement between sides. On the test day, mice were habituated to the test cage twice for 10 min each, prior to behavioural assessment. A 2 × 2 cm filter paper (GE Healthcare, 3030-909), onto which 4 μL of 2,4,5-trimethylthiazole (nTMT; Tokyo Chemical Industry, T1068) had been applied, was placed 40 mm from one end wall and centred laterally. Mouse behaviour was recorded for 10 min using a digital video camera (Nikon). Avoidance time was defined as the duration spent in the compartment opposite to the odour source (the avoidance area). The avoidance index was calculated using the following formula: [(percentage of time spent in the avoidance area during the 10-min test−50)/50]. All tests were conducted after 7:00 p.m. inside a biosafety cabinet, under appropriate containment conditions.

### Olfactory habituation–dishabituation test

The olfactory habituation–dishabituation test (OHDT) (Kobayakawa et al, 2007; Takahashi et al, 2016) was adapted for use under BSL-3 containment conditions. To avoid confounding effects of learning, each mouse was tested only once. Mice were placed in a test cage (TECNIPLAST, 1245ISO00DPBOX), and a filter paper (GE Healthcare, 3030-909; 2 × 2 cm) containing 20 μL of distilled water was presented for 3 min. This presentation was repeated three times with 1-min intervals. On the fourth trial, a filter paper containing 20 μL of 1:1000 diluted eugenol (Nacalai, 15806-42) was introduced for 3 min. Mouse behaviour was recorded using a digital video camera (Nikon), and sniffing behaviour within a 1-cm radius of the odour source was manually scored from video recordings, as previously described (Silverman, Yang et al, 2010; Takahashi et al, 2016). Investigation time was calculated as: (sniffing time during the fourth trial) - (sniffing time during the third trial). In the neutralising antibody treatment experiment, OHDT was conducted at least 8 h after antibody administration on day 13. All tests were performed after 7:00 p.m. inside a biosafety cabinet under appropriate containment conditions.

### Statistics

All data were visualized and analyzed using Prism (v10.4.2). Data were assumed to follow a normal distribution but this was not formally tested. Statistical significance was assessed as follows: Two-way analysis of variance (ANOVA) followed by Sidak’s multiple comparison test was applied to data shown in Figs. 1A, B, D, E, 2B, 2G, 3A, 4F, 5A,C,E, and EV1B. Kruskal–Wallis test followed by Dunn’s multiple comparisons test was used for Fig. 1C. Mann–Whitney test was used for Fig. 1C. Two-way ANOVA followed by Tukey’s multiple comparison test was used for Figs. 1H, 2H, and EV3A. Two-way ANOVA followed by Dunnett’s multiple comparison test was used for Fig. 4A. One-way ANOVA followed by Tukey’s multiple comparison test was applied to Figs. 1B, 4B, C, 5D. Two-tailed unpaired *t* tests were used for Figs. 2C–E, 3B,C, 4D,E,G, 5B, EV2A–D, EV3B, and EV4B,C. *P* values < 0.05 were considered statistically significant. Statistical significance is denoted as follows: *P value < 0.05; **P value < 0.01; ***P value < 0.001; ****P value < 0.0001. Sample sizes were determined based on previous experience with similar experiments in our laboratory. Unless otherwise stated, *n* represents biological replicates.

## Supplementary information

Peer Review File

Source data Fig. 1

Source data Fig. 2

Source data Fig. 3

Source data Fig. 4

Source data Fig. 5

Expanded View Figures

## Data Availability

No primary datasets have been generated and deposited for this study. The source data of this paper are collected in the following database record: biostudies:S-SCDT-10_1038-S44319-026-00769-6.
