# Supplementary figures and images for: Senescence-like cells recruit γδ T cells to drive prolonged hyposmia after SARS-CoV-2 infection in mice
Source: EMBO Rep. 2026 Apr 10;27(10):2526–48. doi: 10.1038/s44319-026-00769-6 (PMC13219478; doi:10.1038/s44319-026-00769-6)

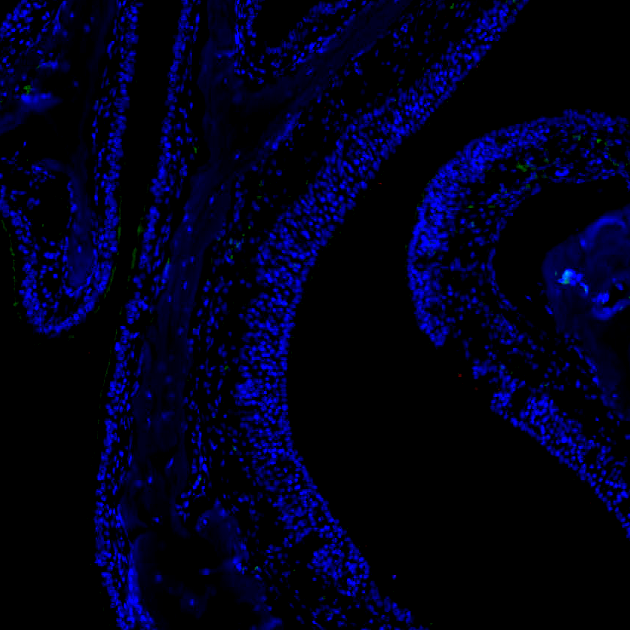

Supplement: Supplementary file 2 — Source data Fig. 1 [file 44319_2026_769_MOESM2_ESM.zip › Figure 1/C/DKO_CoV2_D14.tif]

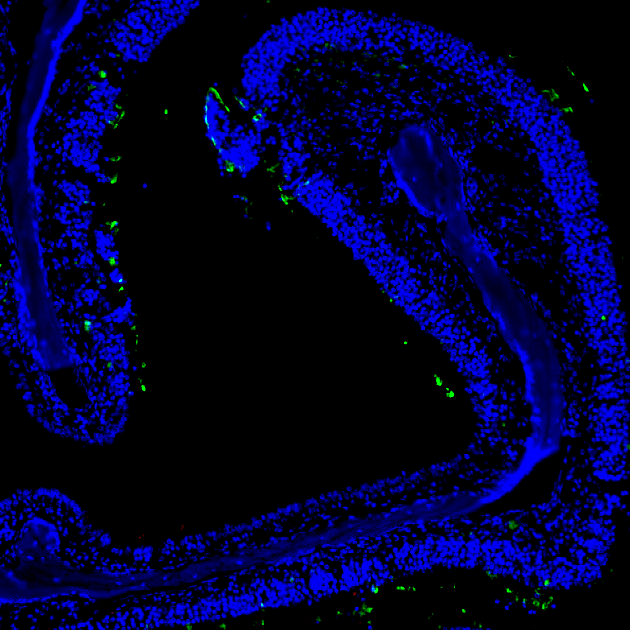

Supplement: Supplementary file 2 — Source data Fig. 1 [file 44319_2026_769_MOESM2_ESM.zip › Figure 1/C/DKO_CoV2_D4.tif]

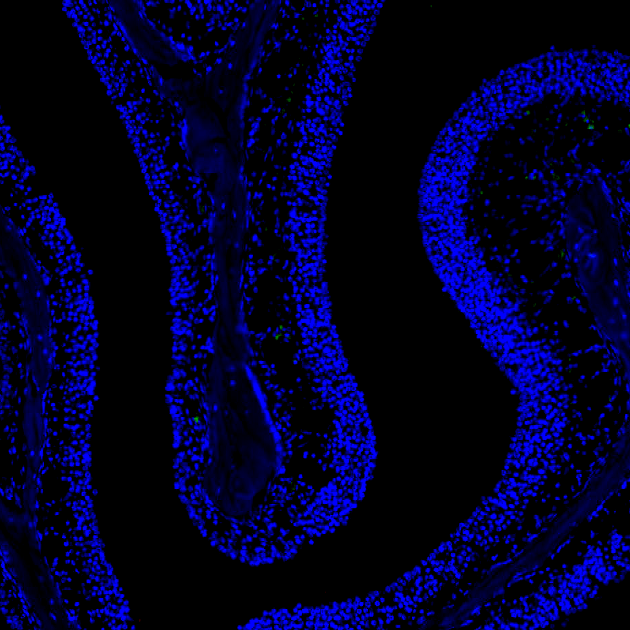

Supplement: Supplementary file 2 — Source data Fig. 1 [file 44319_2026_769_MOESM2_ESM.zip › Figure 1/C/DKO_Mock.tif]

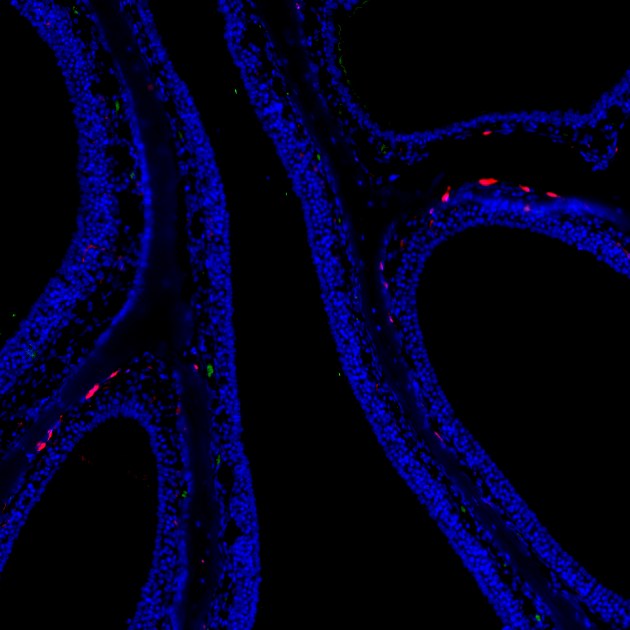

Supplement: Supplementary file 2 — Source data Fig. 1 [file 44319_2026_769_MOESM2_ESM.zip › Figure 1/C/WT_CoV2_D14.tif]

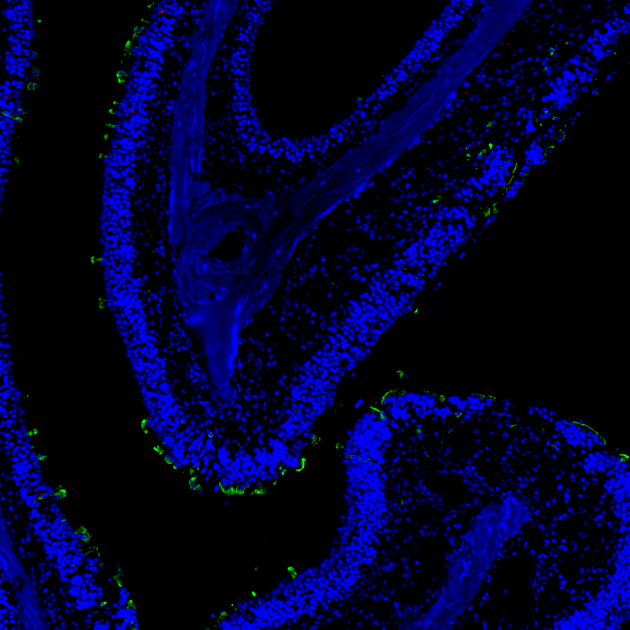

Supplement: Supplementary file 2 — Source data Fig. 1 [file 44319_2026_769_MOESM2_ESM.zip › Figure 1/C/WT_CoV2_D4.tif]

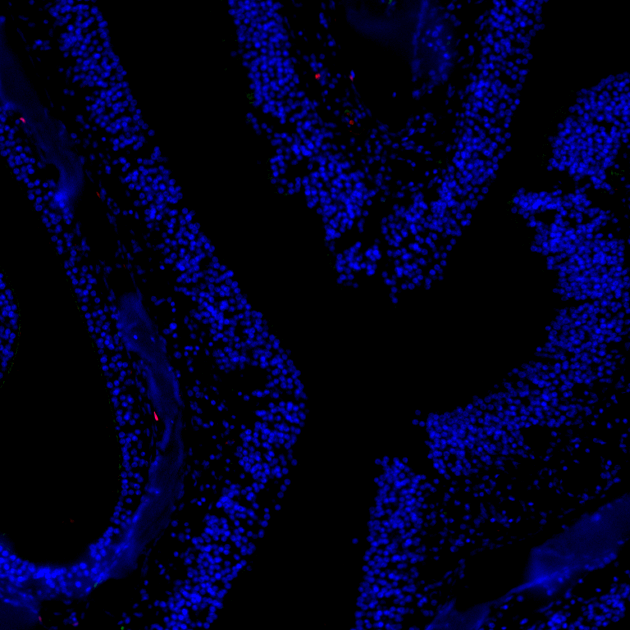

Supplement: Supplementary file 2 — Source data Fig. 1 [file 44319_2026_769_MOESM2_ESM.zip › Figure 1/C/WT_Mock.tif]

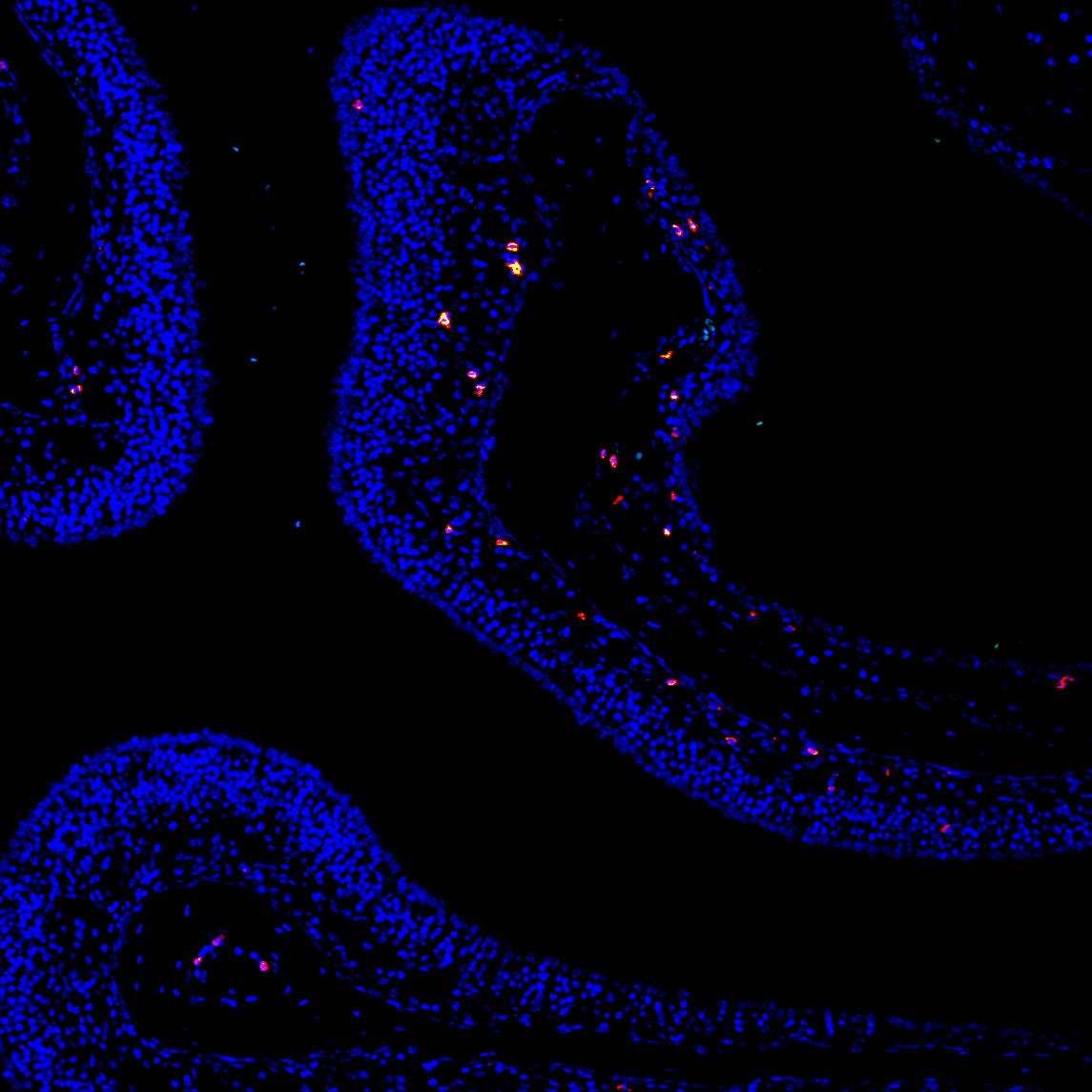

Supplement: Supplementary file 2 — Source data Fig. 1 [file 44319_2026_769_MOESM2_ESM.zip › Figure 1/E/DKO_CoV2.tif]

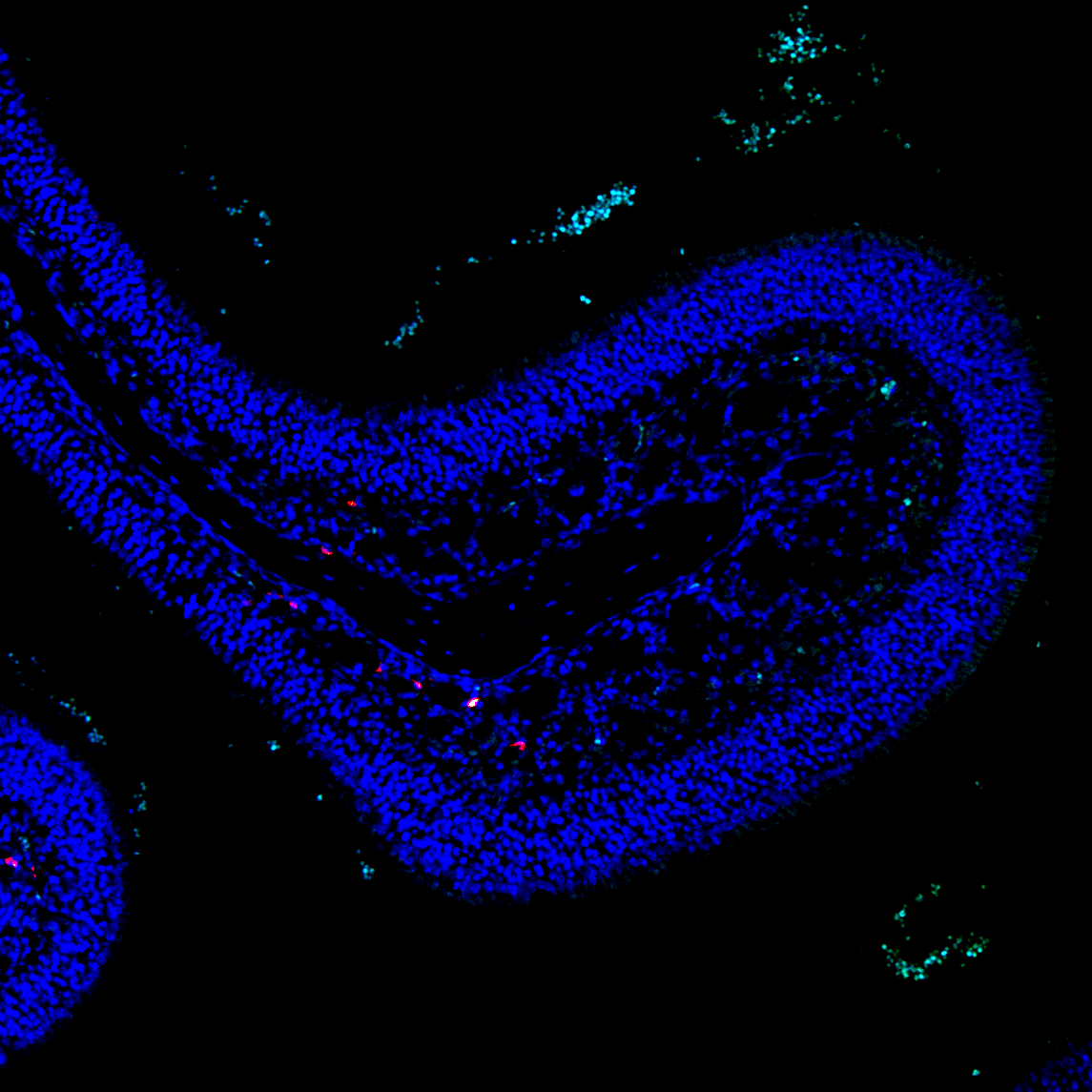

Supplement: Supplementary file 2 — Source data Fig. 1 [file 44319_2026_769_MOESM2_ESM.zip › Figure 1/E/DKO_Mock.tif]

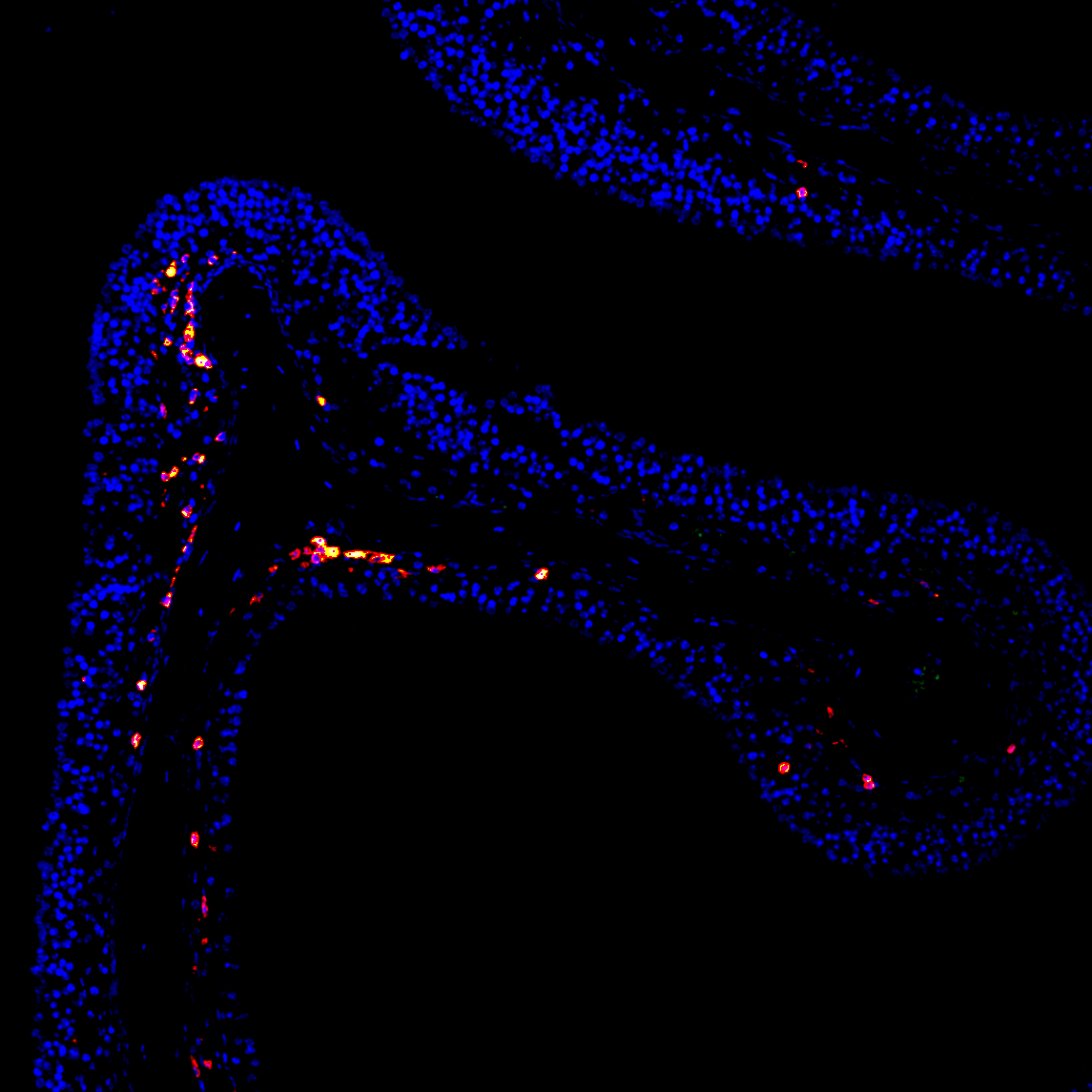

Supplement: Supplementary file 2 — Source data Fig. 1 [file 44319_2026_769_MOESM2_ESM.zip › Figure 1/E/WT_CoV2.tif]

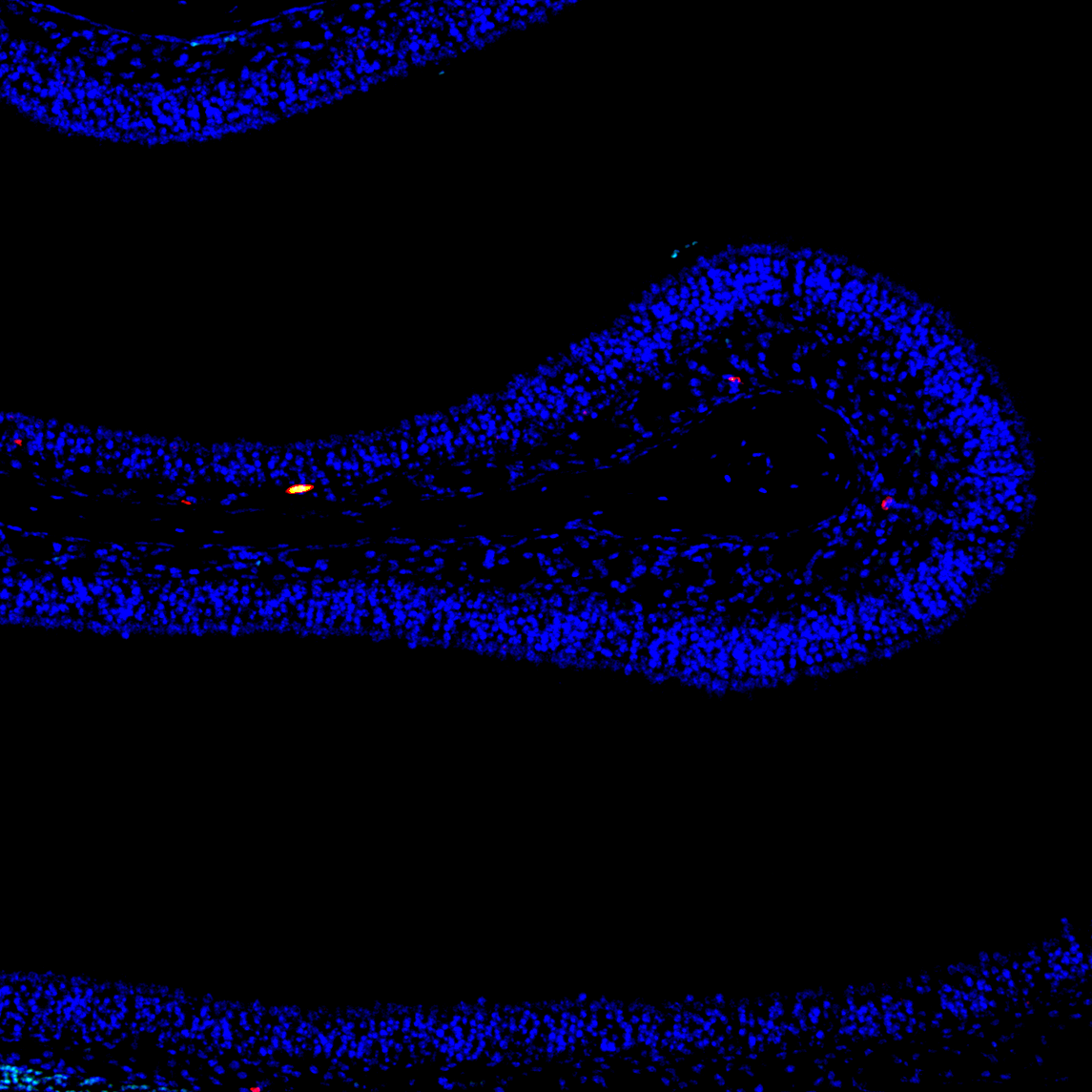

Supplement: Supplementary file 2 — Source data Fig. 1 [file 44319_2026_769_MOESM2_ESM.zip › Figure 1/E/WT_Mock.tif]

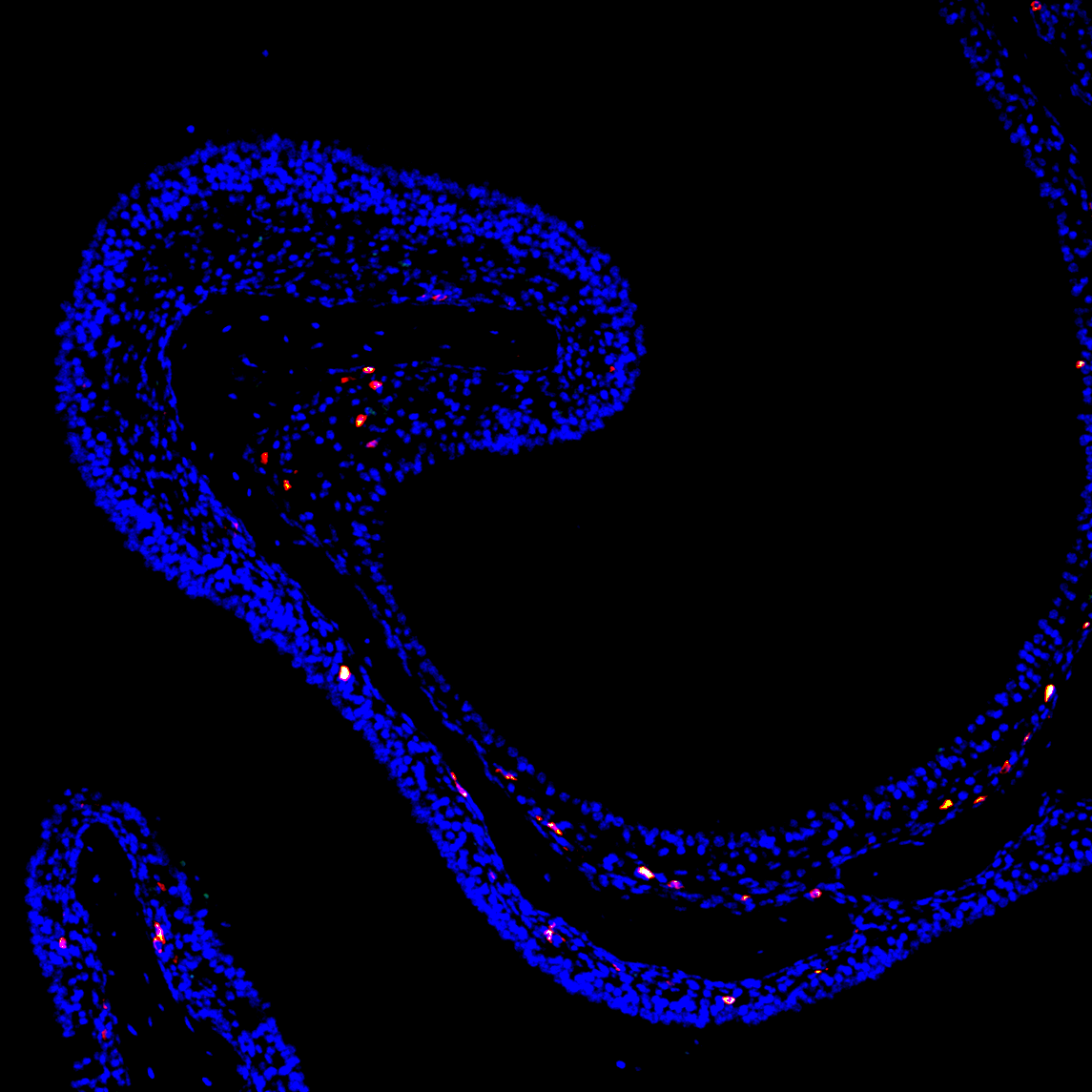

Supplement: Supplementary file 3 — Source data Fig. 2 [file 44319_2026_769_MOESM3_ESM.zip › Figure 2/E/WT_CoV2_ABT.tif]

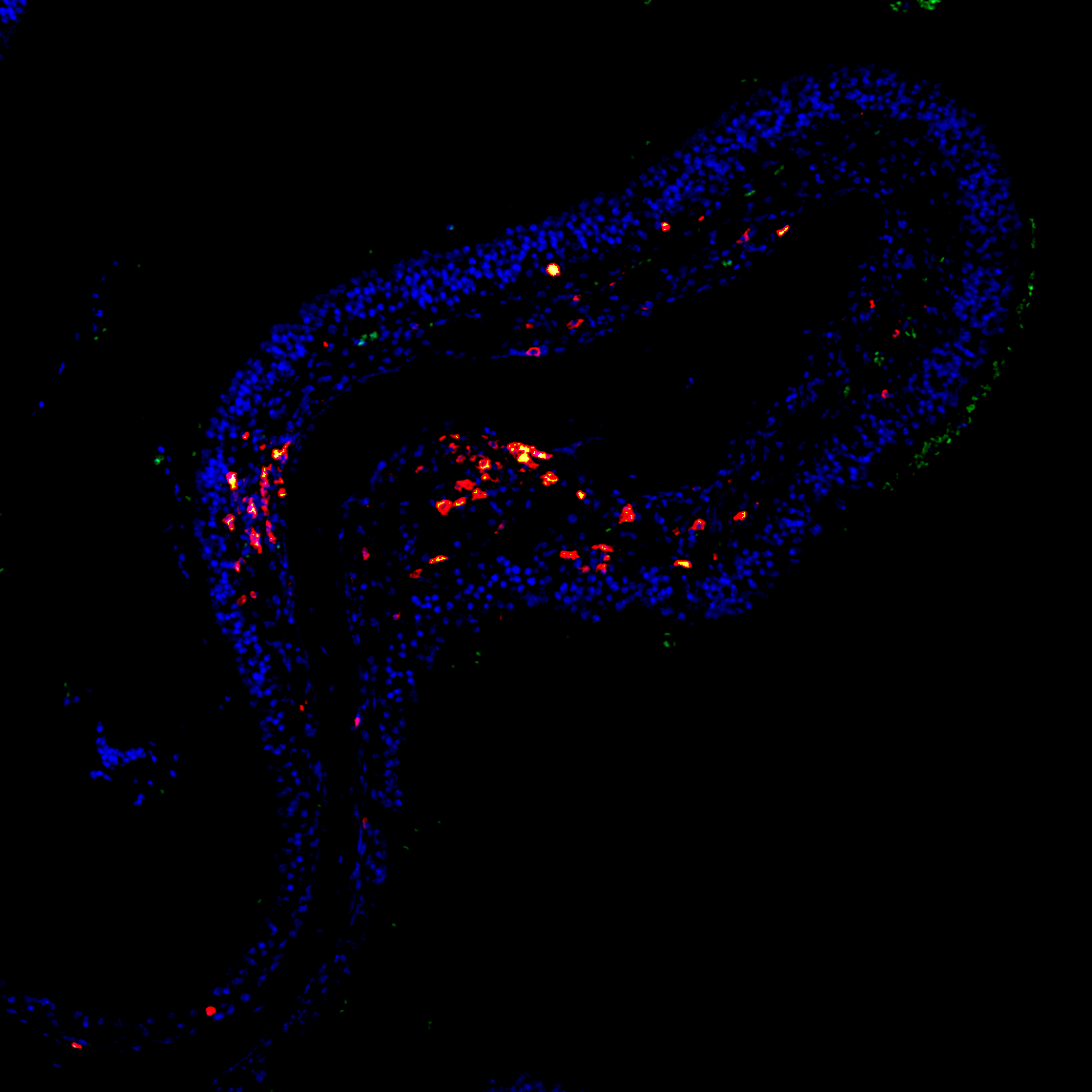

Supplement: Supplementary file 3 — Source data Fig. 2 [file 44319_2026_769_MOESM3_ESM.zip › Figure 2/E/WT_CoV2_Vehicle.tif]

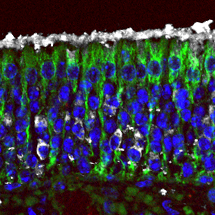

Supplement: Supplementary file 3 — Source data Fig. 2 [file 44319_2026_769_MOESM3_ESM.zip › Figure 2/F/DKO_D14.tif]

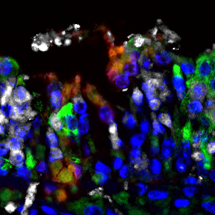

Supplement: Supplementary file 3 — Source data Fig. 2 [file 44319_2026_769_MOESM3_ESM.zip › Figure 2/F/DKO_D4.tif]

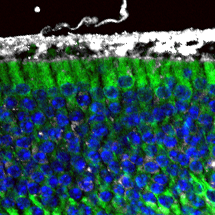

Supplement: Supplementary file 3 — Source data Fig. 2 [file 44319_2026_769_MOESM3_ESM.zip › Figure 2/F/DKO_Mock.tif]

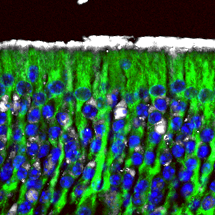

Supplement: Supplementary file 3 — Source data Fig. 2 [file 44319_2026_769_MOESM3_ESM.zip › Figure 2/F/WT_D14.tif]

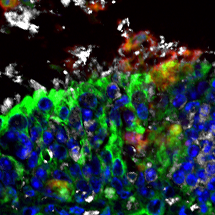

Supplement: Supplementary file 3 — Source data Fig. 2 [file 44319_2026_769_MOESM3_ESM.zip › Figure 2/F/WT_D4.tif]

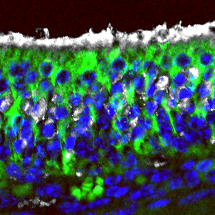

Supplement: Supplementary file 3 — Source data Fig. 2 [file 44319_2026_769_MOESM3_ESM.zip › Figure 2/F/WT_Mock.tif]

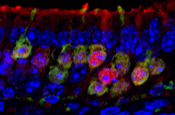

Supplement: Supplementary file 6 — Source data Fig. 5 [file 44319_2026_769_MOESM6_ESM.zip › Figure 5/B/IL17RA^flfl.tif]

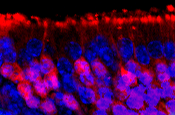

Supplement: Supplementary file 6 — Source data Fig. 5 [file 44319_2026_769_MOESM6_ESM.zip › Figure 5/B/OMP-Cre_IL17RA^flfl.tif]
