# Supplementary material for: Senescence-like cells recruit γδ T cells to drive prolonged hyposmia after SARS-CoV-2 infection in mice
Source: EMBO Rep. 2026 Apr 10;27(10):2526–48. doi: 10.1038/s44319-026-00769-6 (PMC13219478; doi:10.1038/s44319-026-00769-6)
Supplement: Supplementary file 7 — Expanded View Figures [file 44319_2026_769_MOESM7_ESM.pdf]

Expanded View Figures

Figure EV1. A mouse-adapted SARS-CoV-2 strain induces senescence-like phenotypes in human lung diploid fibroblasts (HDFs).

ACE2-HDFs infected with SARS-CoV-2 (MA10 strain; CoV2) or without (Mock) at m.o.i. (multiplicity of infection) of 0.1 were subjected to immunofluorescence analysis (**A**) or to RT–qPCR analysis (**B**) at days 1, 6, and 9. (**A**) Immunofluorescence images of CoV2-NP (red), $p16^{INK4a}$ (green), and DAPI (blue) are shown. The bar graph indicates the percentages of cells expressing CoV2-NP (top) or $p16^{INK4a}$ (bottom). Scale bar, 50 µm. CoV2_NP (*****P* < 0.0001), $p16^{INK4a}$ (Day 1; *ns*; *P* = 0.949, Day 6; *ns*; *P* = 0.804, Day 9; *****P* < 0.0001). (**B**) RT–qPCR analysis of CoV2 RNA, virus- induced cytokines, and SASP factors. Relative mRNA expression levels were determined using the ΔΔCt method after normalization to *GAPDH*. The y-axis shows the relative amount of RNA when the amount of RNA in Mock is set to 1. *Genomic CoV2* (Day 1 vs. Day 6; **P* = 0.0262, Day 6 vs. Day 9; ***P* = 0.0045), *subgenomic CoV2* (Day 1 vs. Day 6; **P* = 0.0431, Day 6 va. Day 9; **P* = 0.0153), *TNFα* (Day 1; *ns*; *P* = 0.997, Day 6; *****P* < 0.0001, Day 9; *ns*; *P* = 0.3915), *IFNβ* (Day 1; *ns*; *P* = 0.988, Day 6; *****P* < 0.0001, Day 9; ***P* = 0.009), *IL6* (Day 1; *ns*; *P* = 0.8785, Day 6; *****P* < 0.0001, Day 9; *ns*; *P* = 0.9449), *IL1β* (Day 1; *ns*; *P* = 0.7162, Day 6; *ns*; *P* = 0.9043, Day 9; ****P* = 0.0086), and *IL8* (Day 1; *ns*; *P* = 0.7802, Day 6; *****P* < 0.0001, Day 9; *****P* < 0.0001). For all graphs, data are presented as mean ± standard deviation (s.d.) of biological triplicate measurements. Statistical significance was determined by two-way ANOVA followed by Sidak's multiple comparison test. *P* values < 0.05 were considered significant. **P* < 0.05, ***P* < 0.01, ****P* < 0.001, *****P* < 0.0001, ns: not significant. N.D. not detected.

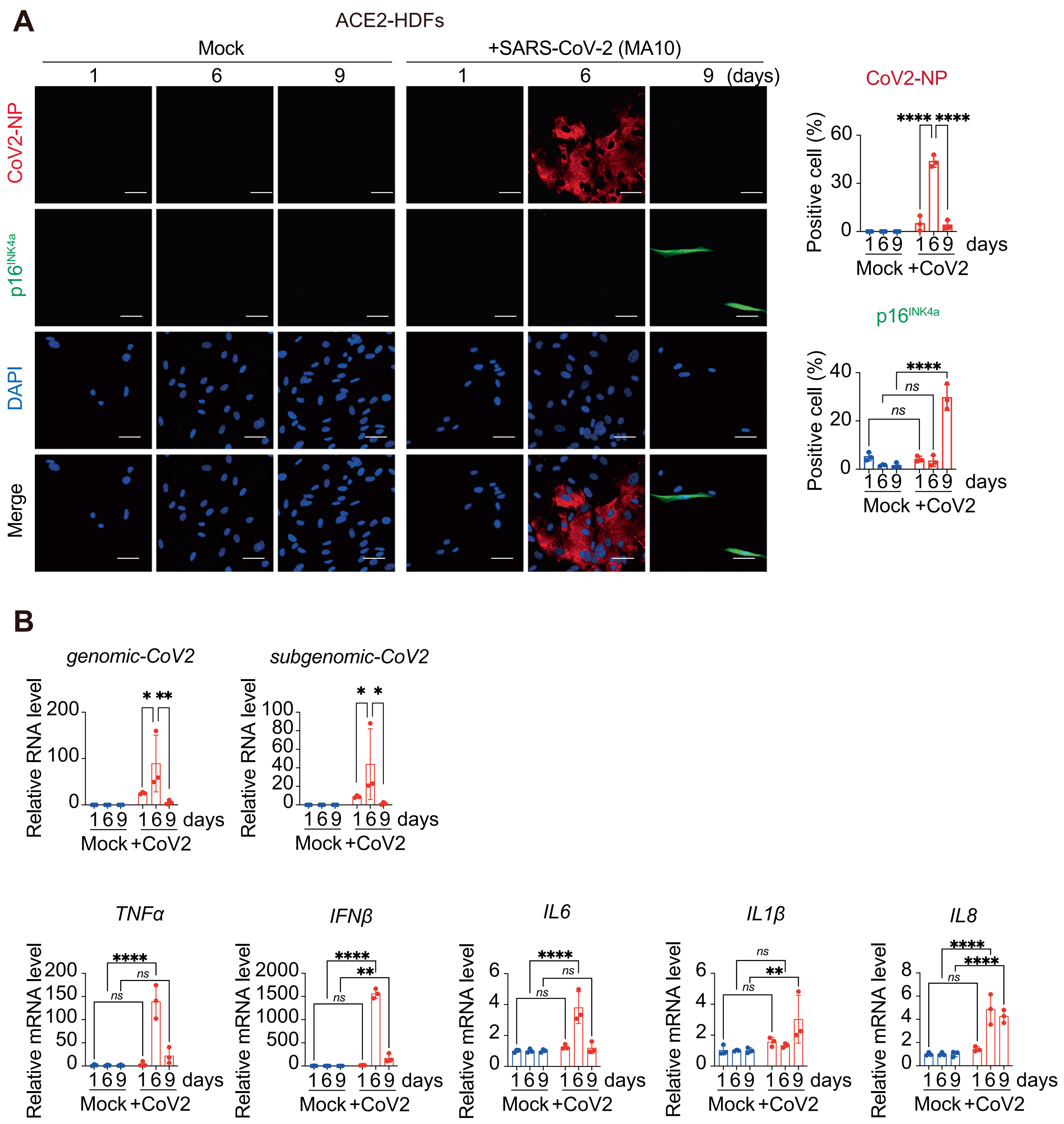
A
ACE2-HDFs
Mock
+SARS-CoV-2 (MA10)
1 6 9 1 6 9 (days)
CoV2-NP
p16INK4a
DAPI
Merge
CoV2-NP
Positive cell (%)
days
Mock +CoV2
p16INK4a
ns
B
genomic-CoV2
subgenomic-CoV2
Relative RNA level
TNFα
IFNβ
IL6
IL1β
IL8
Relative mRNA level

 © The Author(s)

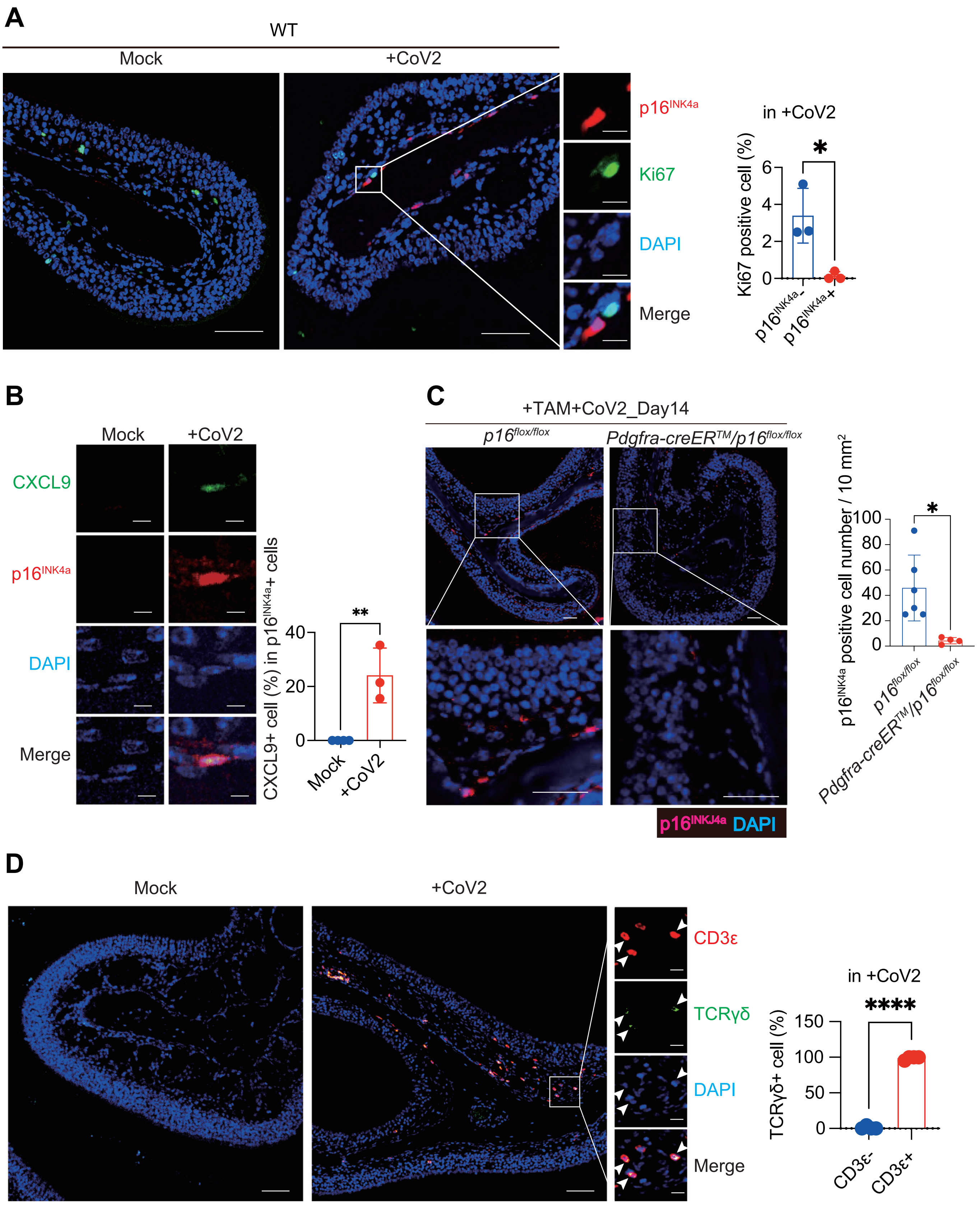
A
WT
Mock
+CoV2
p16INK4a
Ki67
DAPI
Merge
in +CoV2
Ki67 positive cell (%)
*
p16INK4a-
p16INK4a+
B
Mock
+CoV2
CXCL9
p16INK4a
DAPI
Merge
CXCL9+ cell (%) in p16INK4a+ cells
**
Mock
+CoV2
C
+TAM+CoV2_Day14
p16flox/flox
Pdgfra-creERTM/p16flox/flox
p16INKJ4a DAPI
p16INK4a positive cell number / 10 mm2
*
p16flox/flox
Pdgfra-creERTM/p16flox/flox
D
Mock
+CoV2
CD3ε
TCRγδ
DAPI
Merge
in +CoV2
TCRγδ+ cell (%)
****
CD3ε-
CD3ε+

Figure EV2. Appearance of senescence-like fibroblasts and infiltration of γδ T cells in the olfactory mucosa after SARS-CoV-2 infection.

Ten-week-old female wild-type (WT) C57BL/6 mice were intranasally infected with a mouse-adapted strain of SARS-CoV-2 (CoV2). On day 14 post-infection, nasal tissues were collected, fixed in Bouin's solution, and decalcified in 10% EDTA buffer (pH 7.0). After paraffin embedding, 5-μm tissue sections were stained (**A**, **B**). (**A**) Representative images showing Ki67 (green; a marker of proliferating cells), $p16^{INK4a}$ (red), and DAPI (blue). The graph shows the frequency of $Ki67^{+}$ cells among $p16^{INK4a}$-negative and -positive cells located beneath the lamina propria, across the entire field of the olfactory mucosa. Scale bars, 50 μm (overview) and 10 μm (higher magnification). $n = 3$. *n* indicates mice (biological replicates). $^{*}P = 0.0196$. (**B**) Representative images showing CXCL9 (green), p16 (red), and DAPI (blue). The graph shows the percentage of CXCL9-positive cells among total p16-positive cells in the olfactory mucosa. Scale bar: 5 μm. $^{**}P = 0.0044$. (**C**) Fibroblast-specific *p16* knockout mice (*Pdgfra-creER*^{TM}/*p16*$^{flox/flox}$, $n = 4$) and *p16*$^{flox/flox}$ mice ($n = 6$) were treated with tamoxifen (+TAM) and inoculated intranasally with mouse-adapted strain of SARS-CoV-2 (CoV2), when mice were 23 weeks old. On day 14 post-infection, nasal tissues were collected, fixed in Bouin's solution, and decalcified in 10% EDTA buffer (pH 7.0). After paraffin embedding, 5-μm tissue sections were stained for $p16^{INK4a}$ (red) and DAPI (blue). Scale bar, 50 μm. Quantification of $p16^{INK4a}$-positive cells per 10 mm^2 of the olfactory mucosa is shown. $^{*}P = 0.0139$. (**D**) Ten-week-old female wild-type (WT) C57BL/6 mice were intranasally infected with a mouse-adapted strain of SARS-CoV-2 (CoV2). On day 14 post-infection, nasal tissues were collected and stained with antibodies against CD3ε (red) and TCRγδ (green), and counterstained with DAPI (blue) to visualise nuclei. Representative immunofluorescence images demonstrate the co-localization of TCRγδ and CD3ε signals in the olfactory mucosa. Arrowheads indicate $TCR\gamma\delta^{+}$ cells. The accompanying graph shows the frequency of TCRγδ-positive cells among CD3ε-negative and CD3ε-positive cells in the olfactory mucosa. Scale bars, 50 μm (overview) and 10 μm (higher magnification). $n = 4$ mice. $^{****}P < 0.0001$. In all graphs, data represent mean ± standard deviation (s.d.). Statistical significance was determined using an unpaired *t* test. *P* values < 0.05 were considered significant. $^{*}P < 0.05$, $^{**}P < 0.01$, $^{****}P < 0.0001$.

 © The Author(s)

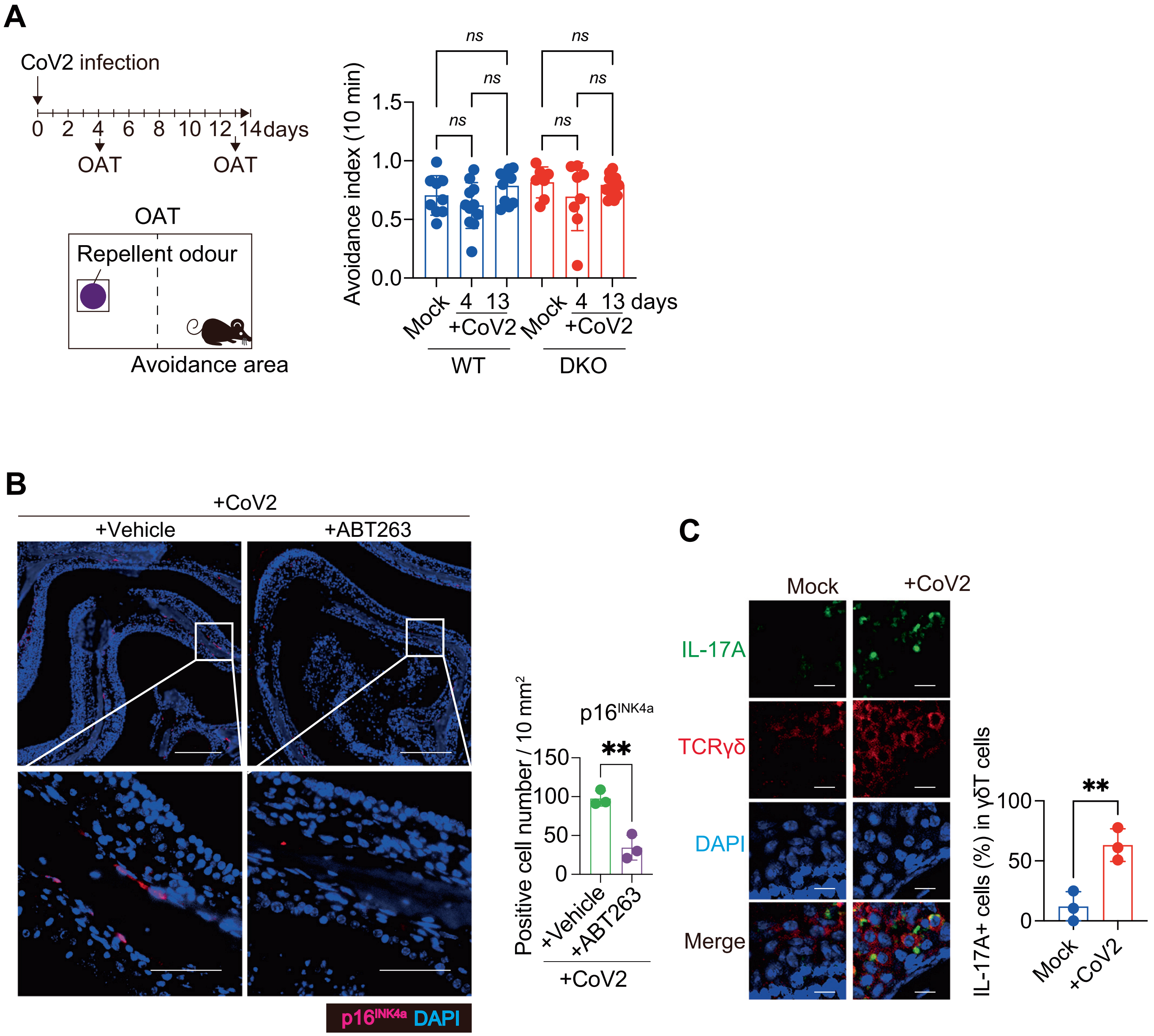

Figure EV3. Olfactory avoidance behavior upon p16⁺ cell depletion and validation of IL-17A-secreting γδ T cells in SARS-CoV-2-infected mice.

(**A**) Ten-week-old female wild-type (WT) C57BL/6 mice or DKO mice were intranasally infected with a mouse-adapted strain of SARS-CoV-2 (CoV2), and an olfactory avoidance test (OAT) was conducted using 2,4,5-trimethylthiazole (nTMT) as a repellent odour. Avoidance behaviour was quantified as the time spent in the half of the cage opposite the odorant compartment (avoidance area). The avoidance index was calculated as: ([percentage of time in avoidance area during 10 min–50]/50). To avoid learning-related bias, each mouse was used only once in the test. Top left: Timeline of the experimental procedures. Bottom left: Schematic diagram of the OAT setup. Right panel: OAT results. Group sizes: WT Mock ($n = 9$), day 4 ($n = 11$), day 13 ($n = 11$); DKO Mock ($n = 7$), day 4 ($n = 8$), day 13 ($n = 12$). n indicates mice (biological replicates). *ns*, WT; $P = 0.8811$ (Mock vs. +CoV2_D4), $P = 0.9041$ (Mock vs. +CoV2_D13), $P = 0.2355$ (+CoV2_D4 vs. +CoV2_D13), DKO; $P = 0.7453$ (Mock vs. +CoV2_D4), $P = 0.9998$ (Mock vs. +CoV2_D13), $P = 0.7989$ (+CoV2_D4 vs. +CoV2_D13). (**B**) Ten-week-old female wild-type (WT) C57BL/6 mice were intranasally infected with a mouse-adapted strain of SARS-CoV-2 (MA10). ABT263 (100 mg/kg) or vehicle was administered by oral gavage at the indicated time points. On day 14 post-infection, nasal tissues were collected, fixed in Bouin's solution, and decalcified in 10% EDTA buffer (pH 7.0). After paraffin embedding, 5 μm tissue sections were stained for $p16^{INK4a}$ (red) and counterstained with DAPI (blue). The graph shows the number of $p16^{INK4a}$-positive cells per 10 mm² of the olfactory mucosa. Scale bars, 200 μm (overview) and 50 μm (higher magnification). $^{**}P = 0.0043$. (**C**) Ten-week-old female wild-type (WT) C57BL/6 mice were intranasally infected with a mouse-adapted strain of SARS-CoV-2 (MA10), and nasal tissues were collected at 14 days post-infection. Representative images showing IL-17A (green), TCRγδ (γδ T cells, red), and DAPI (blue). The graph shows the percentage of IL-17A-positive cells among total TCRγδ-positive cells. Scale bar, 10 μm. $^{**}P = 0.0086$. All data are presented as mean ± standard deviation (s.d.). Statistical significance was determined by two-way ANOVA with Tukey's multiple comparisons test (**A**) or two-tailed unpaired *t* test (**B**, **C**). *P* values < 0.05 were considered significant. $^{**}P < 0.01$, ns: not significant.

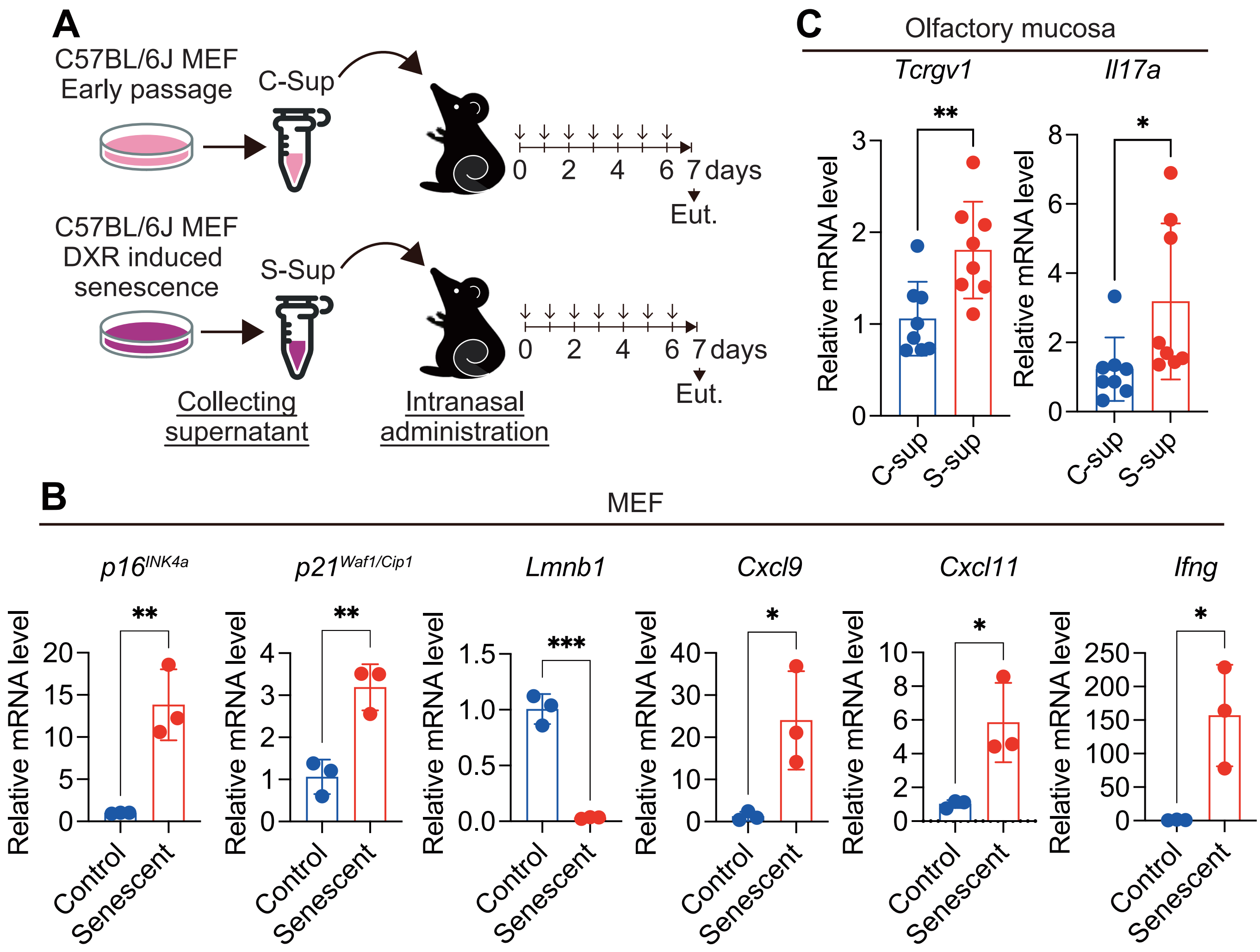

Figure EV4. Intranasal administration of senescent cell supernatant recruits γδ T cells to the olfactory mucosa in mice.

(**A**) Experimental design. Early-passage mouse embryonic fibroblasts (MEFs) derived from wild-type C57BL/6 mice were rendered senescent by treatment with 100 ng/mL doxorubicin (DXR) for 9 days. Afterward, the culture medium was replaced, and senescent MEFs were seeded at 6×10^5 cells per 35-mm dish and incubated for an additional 3 days. Supernatants were then collected (senescent MEF supernatant: S-sup). Control early-passage MEFs (≤6 passages) were seeded at the same density and incubated under identical conditions, and their supernatants were collected (control MEF supernatant: C-sup). Each supernatant (20 µL) was intranasally administered to C57BL/6 mice under anesthesia once daily for 7 consecutive days. Olfactory mucosa was subsequently collected for RT-qPCR analysis. Euthanasia (Eut.) (**B**) RT-qPCR analysis of gene expression in MEFs used for supernatant collection. The expression of senescence markers (*p16*^{INK4a}, *p21*$^{Waf1/Cip1}$, and *Lmnb1*) and SASP-related genes (*Cxcl9, Cxcl11*, and *Ifng*) was quantified using the ΔΔCt method and normalized to 18 s rRNA. qPCRs were performed for 40 cycles. 'Undetermined' data points were assigned a Ct of '40' to enable calculation of fold change. Control, $n = 3$; Senescent, $n = 3$. n indicates mice (biological replicates). *p16*^{INK4a} (**$P = 0.0062$), *p21*$^{Waf1/Cip1}$ (**$P = 0.0057$), *Lmnb1* (***$P = 0.0002$), *Cxcl9* (*$P = 0.0281$), *Cxcl11* (*$P = 0.0238$), and *Ifng* (*$P = 0.0235$). (**C**) RT-qPCR analysis of *Tcrgv1* and *Il17a* expression in the olfactory mucosa of mice intranasally administered MEF supernatants. $n = 8$. Expression levels were calculated using the ΔΔCt method and normalized to β-actin (C-sup set to 1). *Tcrgv1* (**$P = 0.0066$) and *Il17a* (*$P = 0.0392$). All data are presented as mean ± standard deviation (s.d.). Statistical significance was determined using an unpaired *t* test. *P* values < 0.05 were considered significant. *$P < 0.05$, **$P < 0.01$, ***$P < 0.001$, ****$P < 0.0001$.

 © The Author(s)

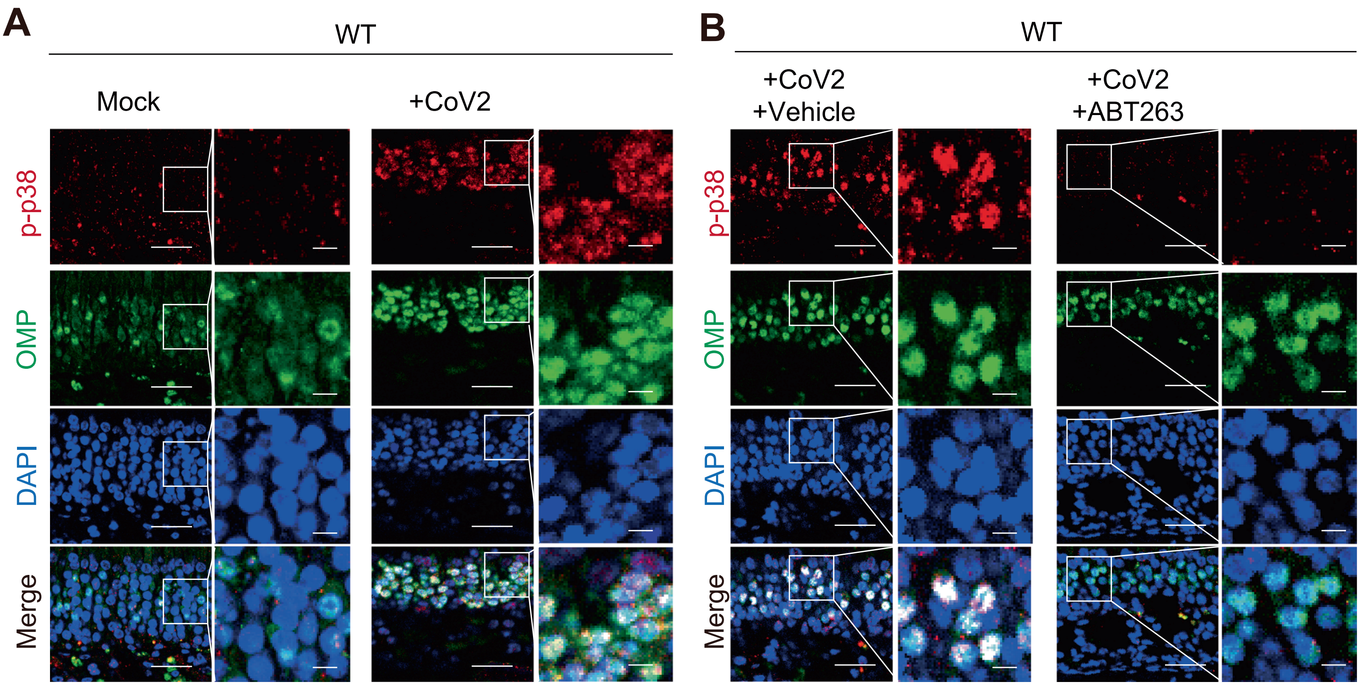

Figure EV5. Senolysis suppresses sustained p38-MAPK activation in olfactory sensory neurons of SARS-CoV-2-infected mice.

Ten- to fourteen-week-old female wild-type (WT) mice were intranasally inoculated with SARS-CoV-2 (MA10; CoV2) and treated with ABT263 or vehicle. The administration schedule for ABT263 is shown in Fig. 2A. All mice were euthanized on day 14 post-infection, and the olfactory mucosa was subjected to immunofluorescence analysis. Representative images showing Phospho-p38 (red), OMP (green), and 4′,6-diamidino-2-phenylindole (DAPI; blue). Areas enclosed by white boxes in each image are shown at higher magnification on the right. Scale bars, 25 μm (low magnification) and 5 μm (high magnification). (**A**) Mock ($n = 3$); +CoV2 ($n = 4$). (**B**) + CoV2 + Vehicle ($n = 5$); +CoV2 + ABT263 ($n = 5$). *n* indicates mice (biological replicates).
