# Supplementary material for: Senescence-like cells recruit γδ T cells to drive prolonged hyposmia after SARS-CoV-2 infection in mice
Source: EMBO Rep. 2026 Apr 10;27(10):2526–48. doi: 10.1038/s44319-026-00769-6 (PMC13219478; doi:10.1038/s44319-026-00769-6)
Supplement: Supplementary file 1 — Peer Review File [file 44319_2026_769_MOESM1_ESM.pdf]

EMBO reports

Senescence-like cells recruit γδ T cells to drive prolonged hyposmia after SARS-CoV-2 infection in mice

Shunya Tsuji, Sosuke Nakano, Koyu Ito, Shohei Minami, Ken Uemura, Yusuke Konishi, Masahiro Wakita, Yumiko Okumura, Shimpei Kawamoto, Akari Matsuki, Shinji Nakaoka, Chikako Ono, Hiroo Takahashi, Itsuki Anzai, Tokiko Watanabe, Akiyoshi Uezumi, Yoshiharu Matsuura, Takeshi Kobayashi, TORU Okamoto, Akio Tsuboi, Masataka Asagiri, and Eiji Hara

Corresponding author(s): Eiji Hara (ehara@biken.osaka-u.ac.jp) , Shunya Tsuji (stsuji@biken.osaka-u.ac.jp), Akio Tsuboi (akio@fbs.osaka-u.ac.jp)

Review Timeline:

Transfer Date:	23rd Jan 26
Editorial Decision:	18th Feb 26
Revision Received:	6th Mar 26
Editorial Decision:	18th Mar 26
Revision Received:	24th Mar 26
Accepted:	26th Mar 26

Editor: Achim Breiling

Transaction Report: Previous rounds of review of this manuscript were performed at another journal.

(Note: With the exception of the correction of typographical or spelling errors that could be a source of ambiguity, letters and reports are not edited. Depending on transfer agreements, referee reports obtained elsewhere may or may not be included in this compilation. Referee reports are anonymous unless the Referee chooses to sign their reports.)

1st Editorial Decision 18th Feb 2026

Dear Prof. Hara,

Thank you for transferring your manuscript that has been reviewed and revised at a journal outside EMBO press to EMBO reports. I now went through the manuscript, and the referee reports and your p-b-p-responses from the previous submissions, and I also discussed the manuscript with my colleagues.

Considering the extensive revisions the manuscript has already undergone and as the referees now agree that the manuscript shows that SARS-CoV-2 infected senescent airway cells promote γδ T-cell accumulation which induces nerve damage and contributes to olfactory dysfunction in mice, we decided to proceed with the study despite the lack of direct human evidence that individuals with persistent post-COVID-19 olfactory dysfunction exhibit a higher senescence burden-or increased γδ T-cell activity-in the olfactory mucosa.

I thus invite you to revise your manuscript and to address the remaining concerns of the two referees (copied again below) as indicated in your revision plan. Please also provide a final p-b-p-response to the referee points with your submission.

- Please indicate already in the title that the data were obtained mainly in mice.

- Please add sub-tiles to the results part, which will render this section more comprehensible.

- Please avoid speculations about therapeutic treatments in the manuscript text (including the abstract).

Revised manuscripts should be submitted within three months of a request for revision. Please contact me to discuss the revision (also by video chat) if you have questions or comments regarding the revision, or should you need additional time.

Moreover, your manuscript also needs to be formatted to our journal style. Please also carefully review the instructions that follow below.

PLEASE NOTE THAT upon resubmission revised manuscripts are subjected to an initial quality control prior to exposition to re-review. Upon failure in the initial quality control, the manuscripts are sent back to the authors, which may lead to delays. Frequent reasons for such a failure are the lack of the data availability section (please see below) and the presence of statistics based on n=2 (the authors are then asked to present scatter plots or provide more data points).

When submitting your revised manuscript, we will require:

1) a .docx formatted version of the final manuscript text (including legends for main figures, EV figures and tables), but without the figures included. Figure legends should be compiled at the end of the manuscript text.

2) individual production quality figure files as .eps, .tif, .jpg (one file per figure), of main figures and EV figures. Please upload these as separate, individual files upon re-submission.

Two main article types are available:

- Research articles, with up to 10 main figures, with additional material provided as Expanded View items (see below) and unlimited references.

- Rports, that are limited to 25,000 characters (excluding spaces, methods, figure legends and references), with up to 5 figures or tables and unlimited references. Expanded View data are permitted but should be restricted to 5 EV figures.

Please decide which format fits best to your finally revised manuscript.

The Expanded View format, which will be displayed in the main HTML of the paper in a collapsible format, has replaced the Supplementary information. For an Article you can submit up to 6 images as Expanded View. Please follow the nomenclature Figure EV1, Figure EV2 etc. The figure legend for these should be included in the main manuscript document file in a section called Expanded View Figure Legends after the main Figure Legends section. Additional Supplementary material should be supplied as a single pdf file labeled Appendix. The Appendix should have page numbers and needs to include a table of content on the first page (with page numbers) and legends for all content. Please follow the nomenclature Appendix Figure Sx, Appendix Table Sx etc. throughout the text, and also label the figures and tables according to this nomenclature.

For more details, please refer to our guide to authors:
https://link.springer.com/journal/44319/submission-guidelines#cms-Revised-submissions

Please consult our guide for figure preparation:

https://media.springernature.com/original/springer-cms/rest/v1/content/27825798/data/v1

3) a .docx formatted letter INCLUDING the reviewers' reports and your detailed point-by-point responses to their comments. As part of the EMBO Press transparent editorial process, the point-by-point response is part of the Review Process File (RPF), which will be published alongside your paper.

4) a complete author checklist, which you can download from our author guidelines (https://link.springer.com/journal/44319/submission-guidelines#cms-Revised-submissions). Please insert page numbers in the checklist to indicate where the requested information can be found in the manuscript. The completed author checklist will also be part of the RPF.

Please also follow our guidelines for the use of living organisms, and the respective reporting guidelines: https://link.springer.com/partners/embo-press/editorial-policies#Animal%20and%20human%20studies

5) that primary datasets produced in this study (e.g. RNA-seq, ChIP-seq, structural and array data) are deposited in an appropriate public database. If no primary datasets have been deposited, please also state this in a dedicated section (e.g. 'No primary datasets have been generated and deposited'), see below.

See also: https://link.springer.com/partners/embo-press/editorial-policies#Data%20deposition

Please remember to provide a reviewer password if the datasets are not yet public.

The accession numbers and database should be listed in a formal "Data Availability" section that follows the model below. This is now mandatory (like the COI statement). Please note that the Data Availability Section is restricted to new primary data that are part of this study. This section is mandatory. As indicated above, if no primary datasets have been deposited, please state this in this section

Data availability

The datasets produced in this study are available in the following databases:

- RNA-Seq data: Gene Expression Omnibus GSE46843 (https://www.ncbi.nlm.nih.gov/geo/query/acc.cgi?acc=GSE46843)
- [data type]: [name of the resource] [accession number/identifier/doi] ([URL or identifiers.org/DATABASE:ACCESSION])

*** Note - All links should resolve to a page where the data can be accessed. ***

See also: https://link.springer.com/partners/embo-press/editorial-policies#Data%20availability%20statement

Moreover, I have these editorial requests:

6) We now request the publication of original source data with the aim of making primary data more accessible and transparent to the reader. You will receive a separate email with instructions for providing source data with your revised manuscript, including information how to upload and organize the files.

7) Our journal encourages inclusion of *data citations in the reference list* to directly cite datasets that were re-used and obtained from public databases. Data citations in the article text are distinct from normal bibliographical citations and should directly link to the database records from which the data can be accessed. In the main text, data citations are formatted as follows: "Data ref: Smith et al, 2001" or "Data ref: NCBI Sequence Read Archive PRJNA342805, 2017". In the Reference list, data citations must be labeled with "[DATASET]". A data reference must provide the database name, accession number/identifiers and a resolvable link to the landing page from which the data can be accessed at the end of the reference. Further instructions are available at: https://link.springer.com/journal/44319/submission-guidelines#cms-Reference-guidelines

8) Regarding data quantification and statistics, please make sure that the number "n" for how many independent experiments were performed, their nature (biological versus technical replicates), the bars and error bars (e.g. SEM, SD) and the test used to calculate p-values is indicated in the respective figure legends (also for EV and Appendix figures). Please also check that all the p-values are explained in the legend, and that these fit to those shown in the figure. Please provide statistical testing where applicable. Please avoid the phrase 'independent experiment' but clearly state if these were biological or technical replicates. Please also indicate (e.g. with n.s.) if testing was performed, but the differences are not significant. In case n=2, please show the data as separate datapoints without error bars and statistics. See also: https://link.springer.com/journal/44319/submission-guidelines#cms-Figure-and-data-presentation

If n<5, please show single datapoints for diagrams.

9) Please add scale bars of similar style and thickness to all microscopic images, using clearly visible black or white bars

(depending on the background). Please place these in the lower right corner of the images themselves. Please do not write on or near the bars in the image but define the size in the respective figure legend.

10) Please also note our reference format:
https://link.springer.com/journal/44319/submission-guidelines#cms-Reference-guidelines

11) We updated our journal's competing interests policy in January 2022 and request authors to consider both actual and perceived competing interests. Please name this section 'Disclosure and Competing Interests Statement' and put it after the Acknowledgements section.
See: https://link.springer.com/partners/embo-press/editorial-policies#Competing%20interest%20disclosures

12) We now use CRediT to specify the contributions of each author in the journal submission system. CRediT replaces the author contribution section. Please use the free text box to provide more detailed descriptions and do NOT provide your final manuscript text file with an author contributions section. See also our guide to authors (section 'Author contributions'):
https://link.springer.com/journal/44319/submission-guidelines#cms-Revised-submissions

13) All Materials and Methods need to be described in the main text using our 'Structured Methods' format, which is required for all research articles. According to this format, the Methods section should include a Reagents and Tools Table (listing key reagents, experimental models, software, and relevant equipment and including their sources and relevant identifiers), uploaded as separate file, and a Methods section in which we encourage the authors to describe their methods using a step-by-step protocol format with bullet points, to facilitate the adoption of the methodologies across labs. More information on how to adhere to this format as well as downloadable templates (.doc) for the Reagents and Tools Table can be found in our author guidelines (section 'Structured Methods'):

https://link.springer.com/journal/44319/submission-guidelines#cms-Manuscript-organisation-and-formatting

Please add all primer or antibody information directly to the Reagents and Tools table.

14) Please order the manuscript sections like this, using only these names:
Title page - Abstract (max. 175 words) - Keywords (max. 5) - Introduction - Results - Discussion - Methods - Data availability section - Acknowledgements (please include here all the funding information) - Disclosure and Competing Interests Statement - References - Figure legends - Expanded View Figure legends

15) Please make sure that all the funding information is also entered into the online submission system and that it is complete and similar to the one in the acknowledgement section of the manuscript text file.

We would also welcome the submission of cover suggestions, or motifs to be used by our Graphics Illustrator in designing a cover.

As part of the EMBO publication's Transparent Editorial Process, EMBO reports publishes online a Review Process File (RPF) to accompany accepted manuscripts. This File will be published in conjunction with your paper and will include the referee reports, your point-by-point response and all pertinent correspondence relating to the manuscript.

You are able to opt out of this by letting the editorial office know (emboreports@embo.org). If you do opt out, the Review Process File link will point to the following statement: "No Review Process File is available with this article, as the authors have chosen not to make the review process public in this case."

I look forward to seeing a revised version of your manuscript when it is ready. Please let me know if you have questions or comments regarding the revision.

Kind regards,

Achim

Achim Breiling
Senior Editor
EMBO Reports

Referee #1

The revised manuscript is strengthened by additional data showing that senescent cells promote γδ T-cell accumulation and contribute to olfactory dysfunction in mice. Both senolytic treatment and IL-17RA deletion support the proposed pathway.

However, a key conceptual limitation remains. The study does not provide direct human evidence that individuals with persistent post-COVID-19 olfactory dysfunction exhibit a higher senescence burden-or increased γδ T-cell activity-in the olfactory mucosa compared with recovered individuals. Because the central model relies on persistent epithelial senescence as the upstream driver of prolonged symptoms, this missing translational link significantly limits the ability to generalize the mouse findings to long-lasting human post-COVID-19 condition.

Current human datasets do not show elevated senescence markers, SASP signatures, or γδ T-cell enrichment in long COVID olfactory tissue, and the manuscript does not present new human data addressing this gap. Without such comparative evidence, it remains unclear whether the mechanism identified in mice reflects a chronic human pathology or primarily explains post-acute, reversible dysfunction.

Referee #3

Tsuji et al. present a second revision their manuscript demonstrating the long-lasting accumulation of senescent cells in mice following intranasal inoculation with a mouse-adapted strain of SARS-CoV-2. The authors have addressed all of my previous concerns, and their reorganization of the extended figures makes the present version of the manuscript easier to follow.

I only have one additional qualm: the authors describe in their response to the reviewers and Extended Figure 8 that they identified hardly any olfactory sensory neurons in the post-covid samples from GSE201620 (Finlay et al. 2022; PMID: 36542694). However, Figure 5c of Finlay et al. clearly shows OSNs from both the post-COVID and normosmic samples (and has UMAP plots showing marker gene expression to demonstrate they are indeed OSNs), so it seems likely that the lack of OSNs is likely due to the analytic approaches by the authors in this manuscript. Similarly, Figure 1 of Finlay et al. shows a large clusters of fibroblasts. Therefore, the authors should double-check the methods and results of their single-cell analyses, consider obtaining the cell-type annotations from these previous papers to help mitigate these discrepancies, or-as previously suggested-consider removing these analyses altogether from the manuscript.

Point-by-point response to Reviewers' comments:

We sincerely thank the Reviewers for their constructive and thoughtful comments, which have been extremely helpful in improving our manuscript. Below, we provide our detailed, point-by-point responses to all comments. Our responses are shown in bold text below. We hope that these revisions and clarifications address all concerns and demonstrate that the revised manuscript merits publication in EMBO Reports.

Reviewer #1 (Remarks to the Author):

The revised manuscript is strengthened by additional data showing that senescent cells promote γδ T-cell accumulation and contribute to olfactory dysfunction in mice. Both senolytic treatment and IL-17RA deletion support the proposed pathway.
However, a key conceptual limitation remains. The study does not provide direct human evidence that individuals with persistent post–COVID-19 olfactory dysfunction exhibit a higher senescence burden—or increased γδ T-cell activity—in the olfactory mucosa compared with recovered individuals. Because the central model relies on persistent epithelial senescence as the upstream driver of prolonged symptoms, this missing translational link significantly limits the ability to generalize the mouse findings to long-lasting human post–COVID-19 condition.
Current human datasets do not show elevated senescence markers, SASP signatures, or γδ T-cell enrichment in long COVID olfactory tissue, and the manuscript does not present new human data addressing this gap. Without such comparative evidence, it remains unclear whether the mechanism identified in mice reflects a chronic human pathology or primarily explains post-acute, reversible dysfunction.

<u>Response:</u>

We sincerely thank the reviewer for the constructive comments and for acknowledging the value of the additional experiments using mouse models.

We fully recognize that the lack of direct human evidence represents an important limitation of our study, as the reviewer rightly points out. However, it is also important to note that, to date, no human dataset exists that contains sufficient numbers of the key cell types central to our findings—fibroblasts, γδ T cells, and

mature olfactory sensory neurons—from age-matched individuals with persistent olfactory dysfunction.

Even in the pioneering scRNA-seq dataset in this field (Finlay et al., *Sci. Transl. Med.*, 2022; GSE201620), we observed notable imbalances in age distribution between healthy controls and post-COVID-19 condition patients, as well as considerable variability in cell-type composition across samples (as documented in Extended Data Fig. 8 of our previous version, which was removed in the current revision). Importantly, currently available human datasets contain very limited numbers of fibroblasts, γδ T cells, and mature olfactory sensory neurons, making it difficult to reliably assess senescence-associated signatures or γδ T-cell enrichment within these specific populations. Therefore, the absence of detectable senescence markers or γδ T-cell signals in existing datasets should be interpreted with caution.

These limitations likely reflect the fact that the olfactory epithelium is confined to anatomically difficult-to-access regions, and that the fibroblasts of interest reside even deeper beneath the lamina propria, making it technically and ethically challenging to consistently obtain sufficient numbers of these cells from human subjects.

In light of these considerations, we have explicitly stated in the revised manuscript that our findings are based on mouse models and that direct human relevance remains to be established, which we now describe as a limitation of the present study (page 12, lines 10–20). We hope that our findings will help motivate future studies using carefully characterized human clinical samples and ultimately contribute to a better understanding of the mechanisms underlying persistent hyposmia in post-COVID-19 condition.

Reviewer #3 (Remarks to the Author):

Tsuji et al. present a second revision their manuscript demonstrating the long-lasting accumulation of senescent cells in mice following intranasal inoculation with a mouse-adapted strain of SARS-CoV-2. The authors have addressed all of my previous concerns, and their reorganization of the extended figures makes the present version of the manuscript easier to follow.

I only have one additional qualm: the authors describe in their response to the reviewers and Extended Figure 8 that they identified hardly any olfactory sensory neurons in the post-covid samples from GSE201620 (Finlay et al. 2022; PMID: 36542694). However, Figure 5c of Finlay et al. clearly shows OSNs from both the post-COVID and normosmic samples (and has UMAP plots showing marker gene expression to demonstrate they are indeed OSNs), so it seems likely that the lack of OSNs is likely due to the analytic approaches by the authors in this manuscript. Similarly, Figure 1 of Finlay et al. shows a large clusters of fibroblasts. Therefore, the authors should double-check the methods and results of their single-cell analyses, consider obtaining the cell-type annotations from these previous papers to help mitigate these discrepancies, or—as previously suggested—consider removing these analyses altogether from the manuscript.

Response:

We thank the reviewer for the constructive comments and sincerely appreciate the continued engagement throughout multiple rounds of review.

We acknowledge that Finlay et al. clearly identified OSN and fibroblast clusters in their analysis, and that OSNs were reported to be present even in post-COVID hyposmia samples. The differences between our initial reanalysis and the annotations reported by Finlay et al. may reflect differences in data processing or analytical approaches.

Regarding fibroblasts, the data presented in Figure 1C of Finlay et al. (*Sci. Transl. Med.*, 2022) include samples not only from healthy controls (non-COVID normosmia) and post-COVID hyposmia patients, but also from an “Other” category (non-COVID hyposmia). As shown in Extended Data Figure 8 of our previous version, while fibroblasts appear to be present in the overall dataset, a closer examination by disease category suggests that most fibroblasts originate from a limited number of samples.

Regarding OSNs, the cells depicted in Figure 5C of Finlay et al. are annotated as “OSN lineage cells,” of which mature OSNs represent only a subset. Our reanalysis confirmed the presence of neuronal cells, but these were derived from only a subset of samples in both post-COVID hyposmia and control groups.

In light of these considerations and in accordance with the reviewer's suggestion, we have removed these analyses from the manuscript. We now explicitly acknowledge the lack of direct human evidence as a limitation of this study (page 12, lines 10–20).

1st Revision - Editorial Decision 18th Mar 2026

Dear Prof. Hara,

Thank you for the submission of your further revised manuscript to our editorial offices. I now went through this and your p-b-p-response and consider the remaining points of the referees as adequately addressed.

Before we can proceed with formal acceptance, I have the editorial requests below I ask you to address in a final revised manuscript. Please also provide a final p-b-p-response to the editorial requests.

Editorial requests:

- Please provide the e-mail addresses for the corresponding authors on the title page. Moreover, we request institutional e-mail addresses for all co-corr. Authors. Thus, please provide an institutional e-mail address for Akio Tsuboi, also in the submission system.

- Please provide the abstract written in present tense throughout.

- Please add up to five keywords to the manuscript and order the manuscript sections like this, using only these names:
Title page - Abstract - Keywords - Introduction - Results - Discussion - Methods - Data availability section - Acknowledgements - Disclosure and Competing Interests Statement - References - Figure legends - Expanded View Figure legends

- It seems you used live virus strains in this study. Please add a paragraph to the methods section (titled 'Biosafety') providing details on where and how biosafety-relevant experiments were performed and that these were approved, and by whom (institution, government).

- Please check again that the number "n" for how many independent experiments were performed, their nature (biological versus technical replicates), the bars and error bars (e.g. SEM, SD) and the test used to calculate p-values is indicated in the respective figure legends (main and EV figures). Please also check that all the p-values are explained in the legend, and that these fit to those shown in the figure. Please provide statistical testing where applicable. Please avoid the phrase 'independent experiment' but clearly state if these were biological or technical replicates. Please also indicate (e.g. with n.s.) if testing was performed, but the differences are not significant. In case n=2, please show the data as separate datapoints without error bars and statistics. See also:

https://link.springer.com/journal/44319/submission-guidelines#cms-Figure-and-data-presentation

If n<5, please show single datapoints for diagrams. Moreover:
- Please note that the exact p values are not provided in the legends of figures 1B, C, D, E, H; 2C, D, E, G, H; 3A-C; 4B-D, F; 5B-E; EV1 A, B; EV2 A-D; EV3 B, C
- Please note that information related to n is missing in the legend of figure EV4 C.

- Please make sure that all microscopic images have scale bars of similar style and thickness, using clearly visible black or white bars (depending on the background). Please place these in the lower right corner of the images themselves. Please do not write on or near the bars in the image but define the size in the respective figure legend. Presently, many scale bars have text nearby.

- The data availability section (DAS) is restricted to information about large datasets generated in the study that have been deposited externally. If no such datasets have been deposited, state this here (e.g. 'No primary datasets have been generated and deposited for this study.'). Please remove all text not related to deposited large datasets from the DAS.

- Please make sure that each figure panel is called out separately and that the panels are called out sequentially. Presently, it seems there are no separate callouts for the panels of Fig. 3. Please check.

- Please add the primer information to the Reagents & Tools Table and remove it from the Methods. Please add callouts for the Reagents & Tools table where applicable. Please also remove the instructions text and the examples from the final Reagents & Tools table.

- In several diagrams you show changes of body weight (e.g. 1A, 2B, 3A, 4A/E and 5A). Please explain in the legend more clearly what is shown (% of what?).

- Please use our reference format (et al needs to be used after 10 author names):
https://link.springer.com/journal/44319/submission-guidelines#cms-Reference-guidelines

- Please make sure that all the funding information is also entered into the online submission system and that it is complete and

similar to the one in the acknowledgement section of the manuscript text file. The grant JP24gm1710004h0003 is mentioned in the manuscript text but not in the submission system. Moreover, the funders provided in the Comments Box need to be removed and provided as separate funders.

In addition, I would need from you uploaded separately:
- a short, two-sentence summary of the manuscript (not more than 35 words).
- two to four short (!) bullet points highlighting the key findings of your study (two lines each).
- a schematic summary figure as separate file that provides a sketch of the major findings (not a data image) in jpeg or tiff format (with the exact width of 550 pixels and a height of not more than 400 pixels) that can be used as a visual synopsis on our website.

I look forward to seeing the further revised version of your manuscript when it is ready.

Best,

Achim Breiling
Senior Editor
EMBO Reports

All minor editorial requests have been addressed by the authors.

Prof. Eiji Hara
The University of Osaka
Institute for Microbial Diseases
3-1, Yamada-oka
Suita
Osaka 565-0871
Japan

Dear Prof. Hara,

Thank you for the submission of your final revised manuscript to our editorial offices. I now went through this and consider the remaining editorial points as adequately addressed.

I thus am very pleased to accept your manuscript for publication in the next available issue of EMBO reports. Thank you for your contribution to our journal.

Your manuscript will be processed for publication by EMBO Press. It will be copy edited and you will receive page proofs prior to publication. Please note that you will be contacted by Springer Nature Author Services to complete licensing and payment information.

You may qualify for financial assistance for your publication charges - either via a Springer Nature fully open access agreement or an EMBO initiative. Check your eligibility: https://link.springer.com/journal/44319/how-to-publish-with-us

Should you be planning a Press Release on your article, please get in contact with embo_production@springernature.com as early as possible in order to coordinate publication and release dates.

If you have any questions, please do not hesitate to contact the Editorial Office. Thank you for your contribution to EMBO Reports.

Yours sincerely,

Achim Breiling
Senior Editor
EMBO Reports

>>> Please note that it is EMBO Reports policy for the transcript of the editorial process (containing referee reports and your response letter) to be published as an online supplement to each paper. If you do NOT want this, you will need to inform the Editorial Office via email immediately. More information is available here: https://link.springer.com/partners/embo-press/editorial-policies#Peer%20review

EMBO Press Author Checklist

Corresponding Author Name: Eiji Hara	
Journal Submitted to: EMBO Reports	
Manuscript Number: EMBOR-2026-63709V1-T	

USEFUL LINKS FOR COMPLETING THIS FORM
The EMBO Journal - Author Guidelines
EMBO Reports - Author Guidelines
Molecular Systems Biology - Author Guidelines
EMBO Molecular Medicine - Author Guidelines

Reporting Checklist for Life Science Articles (updated January
This checklist is adapted from Materials Design Analysis Reporting (MDAR) Checklist for Authors. MDAR establishes a minimum set of requirements in transparent reporting in the life sciences (see Statement of Task: 10.31222/osf.io/9sm4x). Please follow the journal's guidelines in preparing your
Please note that a copy of this checklist will be published alongside your article.

Abridged guidelines for figures
1. Data
The data shown in figures should satisfy the following conditions:
- the data were obtained and processed according to the field's best practice and are presented to reflect the results of the experiments in an accurate and unbiased manner.
- ideally, figure panels should include only measurements that are directly comparable to each other and obtained with the same assay.
- plots include clearly labeled error bars for independent experiments and sample sizes. Unless justified, error bars should not be shown for technical
- if n<5, the individual data points from each experiment should be plotted. Any statistical test employed should be justified.
- Source Data should be included to report the data underlying figures according to the guidelines set out in the authorship guidelines on Data

2. Captions
Each figure caption should contain the following information, for each panel where they are relevant:
- a specification of the experimental system investigated (eg cell line, species name).
- the assay(s) and method(s) used to carry out the reported observations and measurements.
- an explicit mention of the biological and chemical entity(ies) that are being measured.
- an explicit mention of the biological and chemical entity(ies) that are altered/varied/perturbed in a controlled manner.
- the exact sample size (n) for each experimental group/condition, given as a number, not a range;
- a description of the sample collection allowing the reader to understand whether the samples represent technical or biological replicates (including how many animals, litters, cultures, etc.).
- a statement of how many times the experiment shown was independently replicated in the laboratory.
- definitions of statistical methods and measures:
  - common tests, such as t-test (please specify whether paired vs. unpaired), simple χ2 tests, Wilcoxon and Mann-Whitney tests, can be unambiguously identified by name only, but more complex techniques should be described in the methods section;
  - are tests one-sided or two-sided?
  - are there adjustments for multiple comparisons?
  - exact statistical test results, e.g., P values = x but not P values < x;
  - definition of 'center values' as median or average;
  - definition of error bars as s.d. or s.e.m.

Please complete ALL of the questions below.
Select "Not Applicable" only when the requested information is not relevant for your study.

Materials

Newly Created Materials	Information included in the manuscript?	In which section is the information available? (Reagents and Tools Table, Materials and Methods, Figures, Data Availability Section)
New materials and reagents need to be available; do any restrictions apply?	Not Applicable	

Antibodies	Information included in the manuscript?	In which section is the information available? (Reagents and Tools Table, Materials and Methods, Figures, Data Availability Section)
For **antibodies** provide the following information:<br>- Commercial antibodies: RRID (if possible) or supplier name, catalogue number and or/clone number<br>- Non-commercial: RRID or citation	Yes	Reagents and Tools Table

DNA and RNA sequences	Information included in the manuscript?	In which section is the information available? (Reagents and Tools Table, Materials and Methods, Figures, Data Availability Section)
**Short novel DNA or RNA including primers, probes**: provide the sequences.	Yes	Reagents and Tools Table

Cell materials	Information included in the manuscript?	In which section is the information available? (Reagents and Tools Table, Materials and Methods, Figures, Data Availability Section)
**Cell lines:** Provide species information, strain. Provide accession number in repository **OR** supplier name, catalog number, clone number, and/**OR** RRID.	Yes	Reagents and Tools Table, Methods
**Primary cultures:** Provide species, strain, sex of origin, genetic modification status.	Yes	Methods
Report if the cell lines were recently **authenticated** (e.g., by STR profiling) and tested for mycoplasma contamination.	Not Applicable	

Experimental animals	Information included in the manuscript?	In which section is the information available? (Reagents and Tools Table, Materials and Methods, Figures, Data Availability Section)
**Laboratory animals or Model organisms:** Provide species, strain, sex, age, genetic modification status. Provide accession number in repository **OR** supplier name, catalog number, clone number, **OR** RRID.	Yes	Methods
**Animal observed in or captured from the field:** Provide species, sex, and age where possible.	Not Applicable	
Please detail **housing and husbandry conditions**.	Yes	Methods

Plants and microbes	Information included in the manuscript?	In which section is the information available? (Reagents and Tools Table, Materials and Methods, Figures, Data Availability Section)
**Plants:** provide species and strain, ecotype and cultivar where relevant, unique accession number if available, and source (including location for collected wild specimens).	Not Applicable	
**Microbes:** provide species and strain, unique accession number if available, and source.	Yes	Methods

Human research participants	Information included in the manuscript?	In which section is the information available? (Reagents and Tools Table, Materials and Methods, Figures, Data Availability Section)
If collected and within the bounds of privacy constraints report on age, sex and gender or ethnicity for all study participants.	Not Applicable	

Core facilities	Information included in the manuscript?	In which section is the information available? (Reagents and Tools Table, Materials and Methods, Figures, Data Availability Section)
If your work benefited from core facilities, was their service mentioned in the acknowledgments section?	Not Applicable	

Design

Study protocol	Information included in the manuscript?	In which section is the information available? (Reagents and Tools Table, Materials and Methods, Figures, Data Availability Section)
If study protocol has been **pre-registered, provide DOI in the manuscript**. For clinical trials, provide the trial registration number **OR** cite DOI.	Not Applicable	
Report the **clinical trial registration number** (at ClinicalTrials.gov or equivalent), where applicable.	Not Applicable	

Laboratory protocol	Information included in the manuscript?	In which section is the information available? (Reagents and Tools Table, Materials and Methods, Figures, Data Availability Section)
Provide DOI OR other citation details if **external detailed step-by-step protocols** are available.	Not Applicable	

Experimental study design and statistics	Information included in the manuscript?	In which section is the information available? (Reagents and Tools Table, Materials and Methods, Figures, Data Availability Section)
Include a statement about **sample size** estimate even if no statistical methods were used.	Yes	Figures
Were any steps taken to minimize the effects of subjective bias when allocating animals/samples to treatment (e.g. **randomization procedure**)? If yes, have they been described?	Yes	Methods
Include a statement about **blinding** even if no blinding was done.	Yes	Methods
Describe **inclusion/exclusion criteria** if samples or animals were excluded from the analysis. Were the criteria pre-established?<br><br>If sample or data points were omitted from analysis, report if this was due to attrition or intentional exclusion and provide justification.	Yes	Methods
For every figure, are **statistical tests** justified as appropriate? Do the data meet the assumptions of the tests (e.g., normal distribution)? Describe any methods used to assess it. Is there an estimate of variation within each group of data? Is the variance similar between the groups that are being statistically compared?	Yes	Methods, Figure

Sample definition and in-laboratory replication	Information included in the manuscript?	In which section is the information available? (Reagents and Tools Table, Materials and Methods, Figures, Data Availability Section)
In the figure legends: state number of times the experiment was **replicated** in laboratory.	Not Applicable	
In the figure legends: define whether data describe **technical or biological replicates**.	Yes	Figures

Ethics

Ethics	Information included in the manuscript?	In which section is the information available? (Reagents and Tools Table, Materials and Methods, Figures, Data Availability Section)
Studies involving **human participants**: State details of **authority granting ethics approval** (IRB or equivalent committee(s), provide reference number for approval.	Not Applicable	
Studies involving **human participants**: Include a statement confirming that **informed consent** was obtained from all subjects and that the experiments conformed to the principles set out in the WMA Declaration of Helsinki and the Department of Health and Human Services Belmont Report.	Not Applicable	
Studies involving **human participants:** For publication of **patient photos**, include a statement confirming that consent to publish was obtained.	Not Applicable	
Studies involving experimental **animals**: State details of **authority granting ethics approval** (IRB or equivalent committee(s), provide reference number for approval. Include a statement of compliance with ethical regulations.	Yes	
Studies involving **specimen and field samples:** State if relevant **permits** obtained, provide details of authority approving study; if none were required, explain why.	Not Applicable	

Dual Use Research of Concern (DURC)	Information included in the manuscript?	In which section is the information available? (Reagents and Tools Table, Materials and Methods, Figures, Data Availability Section)
Could your study fall under dual use research restrictions? Please check biosecurity documents and list of **select agents and toxins** (CDC): https://www.selectagents.gov/sat/list.htm	Not Applicable	
If you used a select agent, is the security level of the lab appropriate and reported in the manuscript?	Not Applicable	
If a study is subject to dual use research of concern regulations, is the name of the **authority granting approval and reference number** for the regulatory approval provided in the manuscript?	Not Applicable	

Reporting

The MDAR framework recommends adoption of discipline-specific guidelines, established and endorsed through community initiatives. Journals have their own policy about requiring specific guidelines and recommendations to complement MDAR.

Adherence to community standards	Information included in the manuscript?	In which section is the information available? (Reagents and Tools Table, Materials and Methods, Figures, Data Availability Section)
State if relevant guidelines or checklists (e.g., **ICMJE, MIBBI, ARRIVE, PRISMA**) have been followed or provided.	Not Applicable	
For **tumor marker prognostic studies,** we recommend that you follow the **REMARK** reporting guidelines (see link list at top right). See author guidelines, under 'Reporting Guidelines'. Please confirm you have followed these guidelines.	Not Applicable	
For **phase II and III randomized controlled trials**, please refer to the **CONSORT** flow diagram (see link list at top right) and submit the CONSORT checklist (see link list at top right) with your submission. See author guidelines, under 'Reporting Guidelines'. Please confirm you have submitted this list.	Not Applicable	

Data Availability

Data availability	Information included in the manuscript?	In which section is the information available? (Reagents and Tools Table, Materials and Methods, Figures, Data Availability Section)
Have **primary datasets** been deposited according to the journal's guidelines (see 'Data Deposition' section) and the respective accession numbers provided in the Data Availability Section?	Not Applicable	
Were **human clinical and genomic datasets** deposited in a public access-controlled repository in accordance to ethical obligations to the patients and to the applicable consent agreement?	Not Applicable	
Are **computational models** that are central and integral to a study available without restrictions in a machine-readable form? Were the relevant accession numbers or links provided?	Not Applicable	
If publicly available data were reused, provide the respective **data citations in the reference list**.	Not Applicable	
